# Leveraging the shared and opposing genetic mechanisms in the heritable cardiomyopathies

**DOI:** 10.21203/rs.3.rs-8346032/v1

**Published:** 2026-01-27

**Authors:** Daria R. Kramarenko, Poeya Haydarlou, George J. Powell, Joel T. Rämö, Riyad Janan, Claire Prince, Dominic S. Zimmerman, Pantazis Theotokis, Prisca K. Thami, Jan Haas, Sophie Garnier, Frank Rühle, Edwin Poel, Amand F. Schmidt, Sharlene Day, Adam Helms, Rachel Lampert, Victoria Parikh, Jodie Ingles, Iacopo Olivotto, Neal Lakdawala, Anjali Owens, Sara Saberi, John Stendhal, Euan Ashley, Belinda Gray, Mark W. Russell, Thomas D Ryan, Joseph W. Rossano, Dominic Abrams, Erin Miller, Kimberly Lin, Niccolo Maurizi, Alessia Argiro, Colin Berry, Rob Cooper, Andrew S. Flett, Roy S. Gardner, John P. Greenwood, Brian P. Halliday, David Hutchings, Masliza Mahmod, Gerry P. McCann, Stephen P. Page, Charles Peebles, Betty Raman, Peter Swoboda, Amanda Varnava, David Wright, Sanjay Prasad, Stuart Cook, Upsala (Paz) Tayal, Rachel Buchan, Roddy Walsh, Arthur A. M. Wilde, Benjamin Meder, Philippe Charron, Anuj Goel, Ahmad S. Amin, Patrick T. Ellinor, Krishna G. Aragam, Rafik Tadros, Yigal M. Pinto, Carolyn Y. Ho, Hugh Watkins, James S. Ware, Connie R. Bezzina, Sean J. Jurgens

**Affiliations:** 1 Department of Experimental Cardiology, Amsterdam Cardiovascular Sciences, Heart Failure & Arrhythmias, Amsterdam UMC location University of Amsterdam, Amsterdam, the Netherlands; 2 European Reference Network for rare low-prevalence and complex diseases of the heart: ERN GUARD-Heart; 3 DCM-NEXT; 4 National Heart and Lung Institute & MRC Laboratory of Medical Sciences, Imperial College London, London, UK; 5 Cardiovascular Disease Initiative, Broad Institute of MIT and Harvard, Cambridge, MA, USA; 6 Cardiovascular Research Center, Massachusetts General Hospital, Boston, MA, USA; 7 Institute for Molecular Medicine Finland (FIMM), Helsinki Institute of Life Science (HiLIFE), University of Helsinki, Helsinki, Finland; 8 Department of Medicine III, Institute for Cardiomyopathies Heidelberg (ICH), University Hospital Heidelberg, Heidelberg, Germany; 9 Research Unit on Cardiovascular Disorders, Metabolism and Nutrition, Team Genomics and Pathophysiology of Cardiovascular Disease, Sorbone Université, INSERM, Paris, France; 10 ICAN Institute for Cardiometabolism and Nutrition, Paris, France; 11 Bioinformatics Core Facility, Institute of Molecular Biology gGmbH (IMB), Mainz, Germany; 12 Department of Genetic Epidemiology, Institute of Human Genetics, University of Münster, Münster, Germany; 13 Department of Clinical Cardiology, Amsterdam UMC location University of Amsterdam, Amsterdam, the Netherlands; 14 Institute of Cardiovascular Science, Faculty of Population Health, University College London, London, UK; 15 University College London British Heart Foundation Research Accelerator, London, UK; 16 Department of Cardiology, Division Heart and Lungs, University Medical Center Utrecht, Utrecht University, Utrecht, the Netherlands; 17 University of Pennsylvania, Philadelphia, PA, USA; 18 SHaRe; 19 Division of Pediatric Cardiology, University of Michigan, Ann Arbor, MI; 20 Yale School of Medicine, New Haven, CT, USA; 21 Stanford School of Medicine, Stanford, CA, USA; 22 Garvan Institute of Medical Research, Sydney, NSW, Australia; 23 University of Florence, Florence, Italy; 24 Cardiovascular Division, Brigham and Women’s Hospital, Harvard Medical School, Boston, MA, USA; 25 University of Sydney, Sydney, NSW, Australia; 26 Cincinnati Children’s Hospital Medical Center, Cincinnati, OH, USA; 27 Children’s Hospital of Philadelphia & University of Pennsylvania, Philadelphia, PA, USA; 28 Boston Children’s Hospital, Harvard Medical School, Boston, MA, USA; 29 Children’s Hospital of Philadelphia, Philadelphia, PA, USA; 30 University Hospital Lausanne (CHUV), Lausanne, Switzerland; 31 Golden Jubilee National Hospital, Clydebank, Scotland, UK; 32 GoDCM; 33 University of Liverpool / Liverpool Heart and Chest Hospital NHS Foundation Trust, Liverpool, UK; 34 University Hospital Southampton NHS Foundation Trust, Southampton, UK; 35 University of Glasgow, Glasgow, UK; 36 Baker Heart and Diabetes Institute, Melbourne, Australia; 37 Leeds Teaching Hospitals NHS Trust, Leeds, UK; 38 The University of Leeds, Leeds, UK; 39 National Heart and Lung Institute, Imperial College London, London, UK; 40 Royal Brompton and Harefield Hospitals, Guy’s and St. Thomas NHS Foundation Trust, London, UK; 41 University of Manchester, UK; 42 University of Oxford, Oxford, UK; 43 Division of Cardiovascular Sciences, University of Leicester, and the NIHR Leicester Biomedical Research Centre, Glenfield Hospital, Leicester, UK; 44 University of Southampton, Southampton, UK; 45 Imperial College Healthcare NHS Trust, London, UK; 46 MRC Laboratory of Medical Sciences, Imperial College London, London, UK; 47 Cardiovascular and Genomics Research Institute, St George’s University of London, London, UK; 48 Site Heidelberg/Mannheim, DZHK, Heidelberg, Germany; 49 Sorbonne Université, Inserm, Unité de recherche sur les maladies cardiovasculaires et métaboliques, ICAN, F-75013 Paris, France; 50 APHP, Cardiology and Genetics Departments, Pitié-Salpêtrière Hospital, Paris, France; 51 Division of Cardiovascular Medicine, Radcliffe Department of Medicine, University of Oxford, John Radcliffe Hospital, Oxford, UK; 52 Cardiovascular Genomics Initiative, Cleveland Clinic, Cleveland, OH, USA; 53 Electrophysiology Service and Adult Congenital Heart Disease Center, Montreal Heart Institute, Université de Montréal, Montreal, Canada.; 54 Program in Medical and Population Genetics, Broad Institute of Harvard and MIT, Cambridge, MA, USA

## Abstract

Dilated cardiomyopathy (DCM) and hypertrophic cardiomyopathy (HCM) are heart muscle diseases with largely opposing structural and functional phenotypes. Yet, both may lead to the same devastating outcomes of advanced heart failure and life-threatening arrhythmias. Using genome-wide association data from 9,365 DCM cases, 5,900 HCM cases, and over 1.2 million controls, we show that DCM and HCM are largely inversely associated across multiple genomic levels. Modeling both disorders as opposing genetic entities, in case-case GWAS approaches, we identify 100 loci (17 novel) underlying the cardiomyopathy spectrum. Several loci map to potential therapeutic targets (e.g., *ADM*, *CACNA2D2*), and polygenic risk scores derived from these data show strong discrimination between DCM and HCM patients in external datasets (AUC 0.78–0.84; AUPRC ~ 0.85). The pervasive opposing associations suggest that cardiomyocyte-directed therapies may often have opposite effects in DCM versus HCM. Nevertheless, a shared-effect analysis reveals a single locus — near the calcium-buffering gene *CASQ2* — and also identifies a concordant genomic component associated with cardiometabolic health and extracardiac risk factors. By leveraging the shared and opposing genetic mechanisms of DCM and HCM, our work defines the genomic architecture of major cardiomyopathy subtypes and suggests new directions for therapeutics and precision medicine in heart failure.

## Introduction

The heritable cardiomyopathies represent groups of heart muscle diseases with partially overlapping clinical features and genetic causes^1–3^. Cardiomyopathies are among the leading indications for heart transplant, in part due to the limited efficacy associated with most of the available pharmacological therapies. Indeed, most guideline-endorsed treatments aim to offload the heart rather than treat the underlying primary myocardial mechanisms^3,4^. A notable exception is the cardiac myosin inhibitors, which target a key molecular driver of hypertrophic cardiomyopathy (HCM) and have been shown to reduce excessive myosin activity and improve symptoms in HCM patients with obstruction^5,6^. Nevertheless, such therapies are not yet indicated in other HCM patients^7,8^, and similar myocardial mechanism-informed therapies are largely unavailable for other heritable cardiomyopathies, including dilated cardiomyopathy (DCM)^9^.

It is well-established that rare genetic variation contributes to both DCM and HCM, with several Mendelian genes - particularly those encoding sarcomere proteins - shared across both cardiomyopathies^10,11^. Functional studies have subsequently demonstrated that DCM- and HCM-causing sarcomere variants exert opposing effects on cardiomyocyte mechanics and contractility^12–14^. More recently, large genome-wide association studies (GWAS) have shown that common genetic variants also contribute substantially to the risk of both DCM and HCM^15–17^, and have highlighted a substantial overlap in common variant loci across both cardiomyopathies, most of which show inverse effects on risk of DCM and HCM^18^. For instance, specific cardiomyopathy risk loci, including the *BAG3 locus*, may modulate penetrance for DCM and HCM in opposite directions of effect^19^. Similarly, a polygenic risk score for HCM, built from GWAS data, was shown to predict a lower risk of DCM^20^. Indeed, despite both leading to similar adverse outcomes - including arrhythmia, heart failure, and sudden death - DCM and HCM display partially opposing phenotypic characteristics.

**Figure 1:**
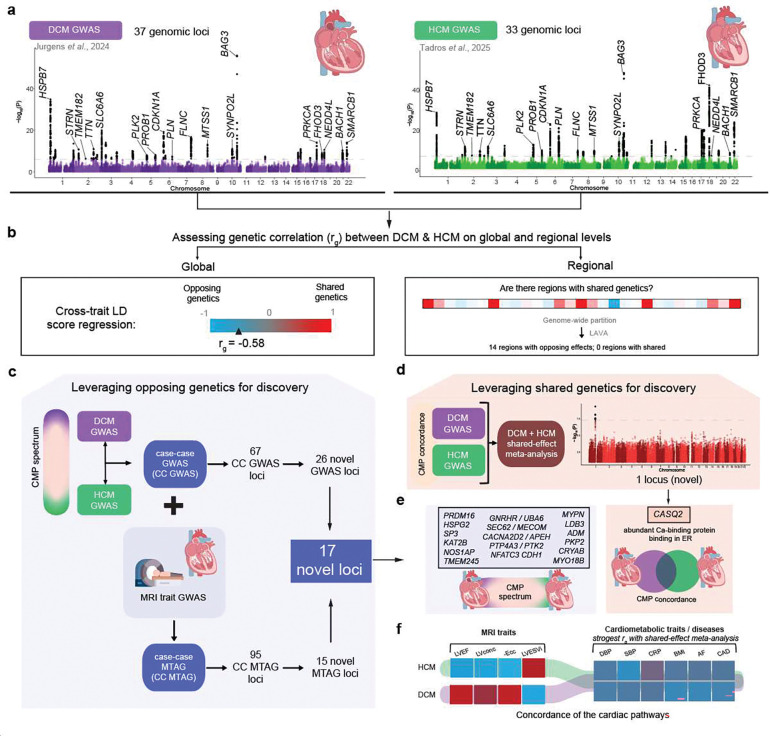
Study overview. **a.** Two Manhattan plots recreated from summary statistics of previously published case-control GWAS for DCM^15^ (37 loci; on the left) and HCM^16^ (33 significant loci; on the right). The plots highlight 18 loci with annotated genes shared between the two studies. **b**. Assessment of the genetic correlation between DCM and HCM on global and regional levels. Left plane: global genetic correlation was assessed using bivariate LD score regression (LDSC)^22^, which highlighted a strong inverse genetic correlation between DCM and HCM. Right plane: regional genetic correlations were evaluated across genome-wide regions using LAVA^23^, identifying 14 regions with significant inverse genetic effects between DCM and HCM. **c.** To model DCM and HCM as opposites on a single genetic spectrum, a statistical case-case GWAS (CC-GWAS) approach was implemented^24^; CC-GWAS was further combined with GWAS data for cardiac MRI traits in an MTAG^41^. These CC-GWAS approaches identified dozens of novel loci for the cardiomyopathy spectrum. **d.** To model DCM and HCM as similar diseases, a shared-effect meta-analysis was applied, which identified one novel locus. **e.** Contemporary locus-to-gene mapping was used to unveil novel genes underlying the cardiomyopathy spectrum. **f**. Genetic concordance of cardiac pathways across MRI traits and cardiometabolic phenotypes. Visualization of genetic correlations (*r*_g_) between DCM and HCM GWAS and a range of quantitative cardiac MRI traits and cardiometabolic diseases. Opposing correlations for DCM and HCM across ventricular function traits reflect their placement at opposite ends of the cardiomyopathy spectrum. In contrast, concordant associations with several cardiometabolic traits highlight shared biological pathways. Traits showing the strongest alignment with both cardiomyopathies were identified using the shared-effect meta-analysis.

Motivated by these findings, we aimed to deeply interrogate the shared and opposing genetic mechanisms underlying DCM and HCM. We hypothesized that the genetically correlated architecture between the two cardiomyopathies could be exploited, using joint analytical frameworks, to identify novel cardiomyopathy loci relevant to mechanistic understanding, therapeutic targeting, and genetic risk prediction (Figure 1).

## Results

### Dissecting the global genetic correlation between DCM and HCM

To investigate the genetic similarity between both cardiomyopathies, we utilized summary-level data from the latest GWAS meta-analyses for HCM^16^ (N=5,900 cases) and DCM^15^ (N=9,365 cases). To ensure consistency and data quality, all summary statistics were reprocessed to include only high-quality common variants. Among other filters, we restricted to variants with adequate sample size and removed the genomic region surrounding *MYBPC3*, a region tagging rare founder pathogenic variants in HCM^16,20^. In addition, we applied a harmonized pipeline for locus definition, fine-mapping, and gene prioritization, to ensure consistency across traits and analytical approaches (Methods, **Supplementary Table 1**). Notably, across all GWAS datasets, gene prioritization was based on the FLAMES framework^21^, a pipeline that integrates multiple layers of biological evidence and uses a machine-learning algorithm to assign prioritization scores to candidate effector genes. Using these definitions, we identified 37 and 33 genome-wide significant loci in DCM GWAS and HCM GWAS, respectively, many of which mapped to plausible effector genes (**Supplementary Table 2**).

**Figure 2:**
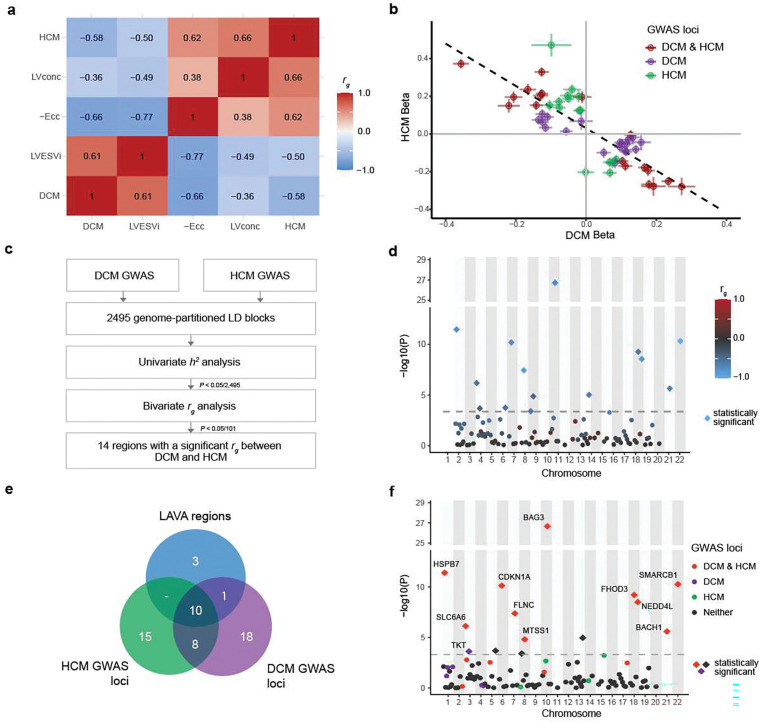
Global and regional genetic correlations between DCM and HCM. **a** is a heatmap of bivariate genetic correlations estimated from GWAS data, showing DCM (on the left), HCM (on the right), and relevant cardiac MRI traits in between, including negative global circumferential strain (-Ecc), left ventricular end-systolic volume (LVESVi), and left ventricular concentricity (LVconc). The color represents the level of genetic correlation, with red and blue representing positive and negative correlation, respectively. (**Supplementary Table 3**) Panel **b** is a dot plot showing effects of lead variants from DCM and HCM GWAS, with estimated effects on DCM on the x-axis and effects on HCM on the y-axis. Loci are colored depending on whether they reached genome-wide significance in DCM GWAS (purple), HCM GWAS (green), or both (red). Error bars represent standard errors. (Extended Data Figure 1; **Supplementary Table 4**) Panel **c** shows an overview of the steps used to analyze regional bivariate genetic correlations between DCM and HCM, using LAVA. Panel **d** is a Manhattan plot showing results from LAVA, where each genomic region is represented by a single dot with genomic regions on the x-axis and the −log10 of the *P*-value on the y-axis. The color represents the level of genetic correlation, with red and blue representing positive and negative correlation, respectively. Panel **e** is a Venn diagram showing the overlap of genome-wide significant DCM loci, significant HCM loci, and significant regions from LAVA. Panel **f** is the same Manhattan plot from panel **d**, now highlighting in color whether the regions overlap significant loci from DCM GWAS (purple), HCM GWAS (green), both (red), or neither (black). (**Supplementary Table 5)** Since Ecc and Ell are always negative values, -Ecc and -Ell are displayed to facilitate interpretation of effect direction.

We first assessed the genetic correlation between DCM and HCM on the global level. Using bivariate LD score regression^22^, we identified a strong negative genetic correlation between DCM and HCM (*r*_g,global_=−0.58, SE = 0.054, *P*=7.3×10^−27^). Consistently, we identified inverse patterns of genetic correlation with relevant left ventricular (LV) endophenotypes from MRI^16^. For instance, DCM showed negative genetic correlation with contractility parameters (eg, negative global circumferential strain (-Ecc): *r*_g,global_=−0.66, *P*=3.7×10^−36^) and positive genetic correlation with indexed LV end-systolic volume (LVESVi)^21^(*r*_g,global_=0.61, *P*=3.5×10^−31^). In contrast, HCM showed a strong positive correlation with LV concentricity (*r*_g,global_=0.66, *P*=2.7×10^−31^), as well as a positive genetic correlation with contractility parameters and negative genetic correlation with LVESVi (Figure 2a; **Supplementary Table 3**). Conditional genetic correlation analyses further supported a shared genetic architecture through MRI traits (**Supplementary Note**). These findings highlight a largely opposing genetic architecture between DCM and HCM, consistent with previous findings from smaller datasets^18^.

### Assessing local and regional genetic correlations

**Extended Data Figure 1:**
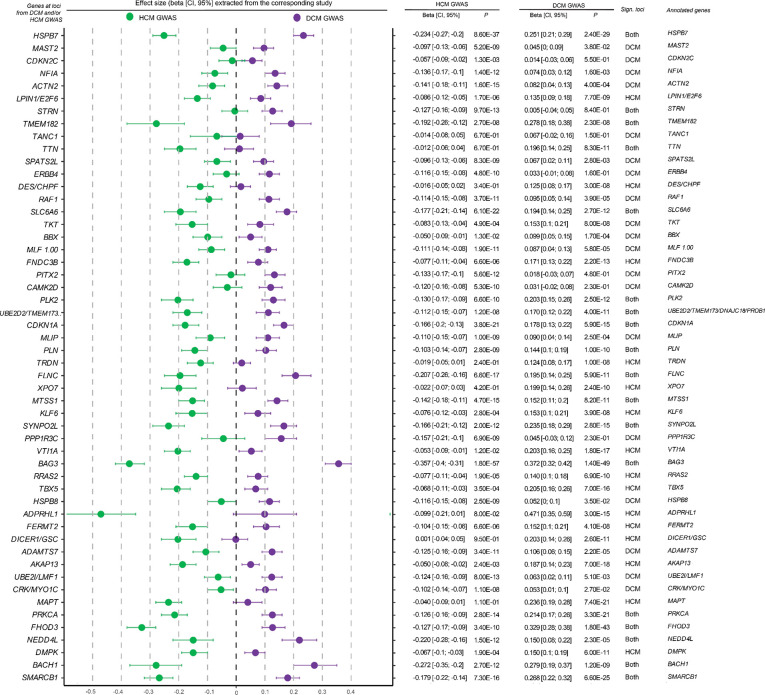
Forest plot of lead SNPs from DCM and HCM GWAS loci Effect sizes and 95% confidence intervals for lead variants at each locus are shown based on association HCM GWAS (green, left) and DCM GWAS (purple, right). Each point represents the estimated effect size (β) from the corresponding study, with horizontal bars indicating the 95% confidence interval. Loci are annotated to candidate genes using integrative locus-to-gene mapping approaches, and the significance of associations is indicated per trait. (Methods) To enable direct visual comparison of directionality, effect sizes, and alleles, the data were aligned across traits. Specifically, for each overlapping locus, we aligned effect sizes such that HCM effects were always visualized on the left and DCM effects on the right. To achieve this, we compared the direction of effect between traits and, if the DCM effect size was smaller than the HCM effect size, we flipped the effect alleles and corresponding β estimates. This harmonization step ensured that the more positive effect consistently appeared on the left (HCM) and the more negative or opposing effect on the right (DCM), facilitating direct visual comparison. Variant identifiers (CHR:POS:A1:A2 format) were updated accordingly to reflect the harmonized allele orientation (original betas in **Supplementary Table 4**, visualized in Figure 2b).

Having identified a strong inverse genetic relationship between DCM and HCM on the global level, we then aimed to assess the genetic correlation on a finer genomic scale. The inverse genetic relationship was notable within specific genomic loci: Across DCM GWAS (37 loci) and HCM GWAS (33 loci), we identified a total of 52 distinct genome-wide significant loci, of which 18 overlapped. Consistent with recent findings^18,19^, all lead variants at 18 overlapping loci — and 97% of the variants that were genome-wide significant in only one GWAS — exhibited evidence of opposing effects between the two cardiomyopathies (Figure 2b; Extended Data Figure 1; **Supplementary Table 4**).

To further investigate regional genetic overlap and identify additional regions with potential opposing or concordant effects, we analyzed 2,495 genome-wide partitions using Local Analysis of Variant Association (LAVA)^23^. Within the LAVA framework (Figure 2c), we first estimated regional heritabilities (*h*^2^_regional_), retaining regions with significant *h*^2^_regional_ for both DCM and HCM (*P*<0.05/2,495). These regions were then tested for genetic correlation (*r*_g,regional_) using bivariate analysis. After Bonferroni correction for the number of tested regions (*P*<0.05/101), we identified 14 genomic regions with significant genetic correlation, all with opposing genetic effects (Figure 2c–d; **Supplementary Table 5**, Methods).

Of the 14 significant LAVA regions, 10 overlapped with loci that were genome-wide significant in both HCM GWAS and DCM GWAS (Figure 2e–f). The regions that reached the highest significance in the regional genetic correlation analysis — near *BAG3*, *FHOD3*, *CDKN1A*, *SMARCB1*, and *HSPB7* — overlap the most strongly associated GWAS loci^15,16,18^, and map to various contractile and non-contractile proteins (**Supplementary Table 5**). In addition to these shared loci, LAVA identified one region uniquely discovered in DCM GWAS (i.e., the locus did not reach significance in HCM GWAS; near *TKT*), as well as three regions not captured by either GWAS (Figure 2f, **Supplementary Note**). These findings indicate that additional cardiomyopathy loci, with inverse effects, remained unidentified in these case-control GWAS.

### Leveraging opposing genetics for discovery

**Figure 3:**
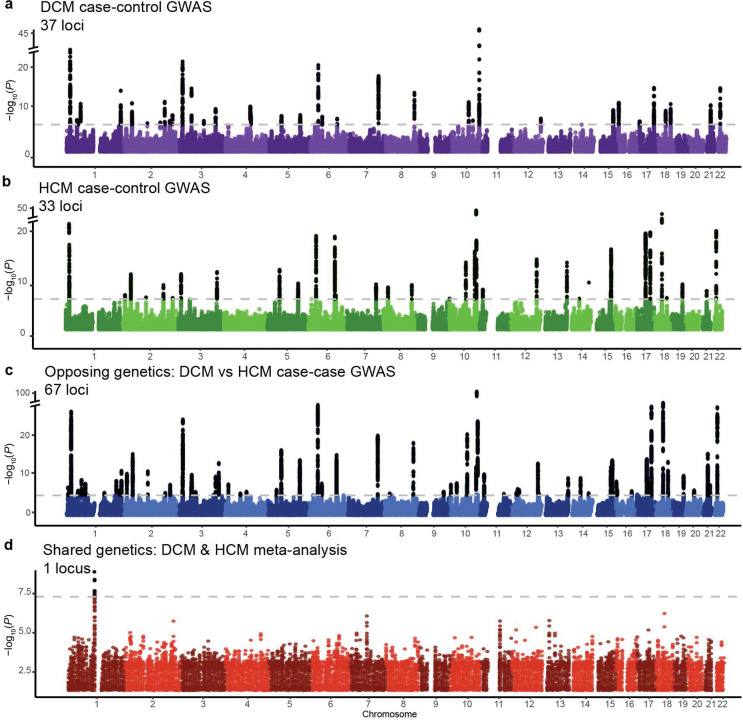
Modeling DCM and HCM as opposites on a disease spectrum yields improved locus discovery. All panels are Manhattan plots where each dot represents a genetic variant, with genomic positions on the x-axis and −log10 of the association *P*-value on the y-axis. Panels **a** and **b** show results for published DCM GWAS (37 loci) and HCM GWAS (33 loci), respectively. Panel **c** shows results from a case-case GWAS in which DCM and HCM are modeled as opposites on a single genetic spectrum, yielding 67 significant loci. Panel **d** shows results for a shared-effect meta-analysis in which DCM and HCM are treated as similar diseases, yielding only one significant locus.

**Figure 4:**
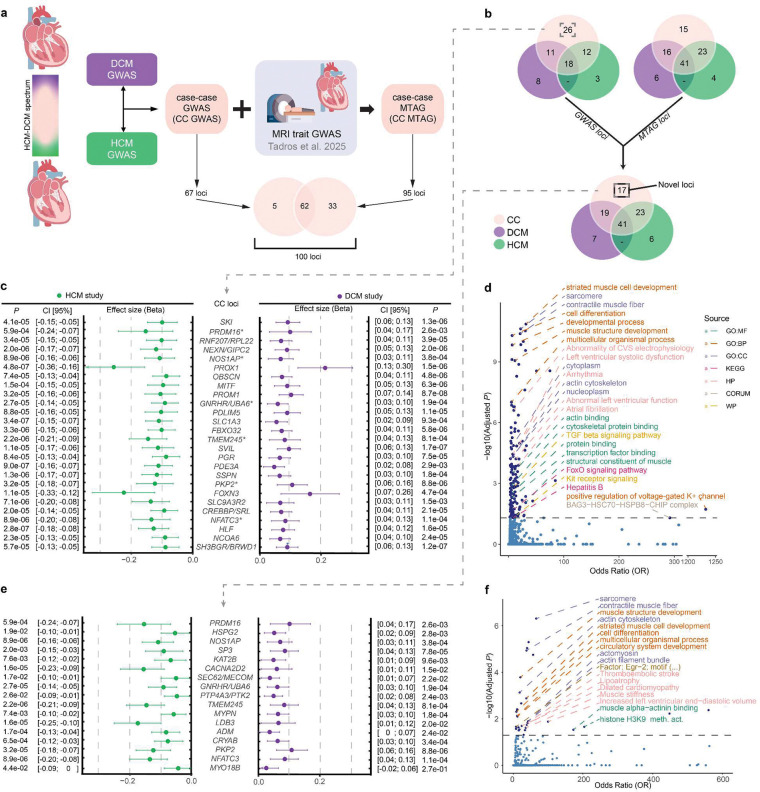
Case-case approaches identify novel genetic targets and highlight pervasive inverse myocardial pathways underlying DCM and HCM. Panel **a** is a schematic illustrating the approach to CC-GWAS and a subsequent multi-trait approach (MTAG) in which CC-GWAS was analyzed jointly with relevant cardiac MRI traits (CC-MTAG). CC-GWAS identified 67 genome-wide significant loci, while CC-MTAG identified 95 significant loci (100 unique loci across both approaches; Extended Data Figure 3). Panel **b** shows various Venn diagrams highlighting the overlap between genome-wide significant loci identified from DCM GWAS/MTAG (Jurgens et al.; purple), HCM GWAS/MTAG (Tadros et al.; green), and our case-case approaches (CC; beige). The left diagram shows overlap for GWAS loci (ie, non-MTAG loci), the right diagram shows overlap for MTAG loci, and the center-bottom diagram shows overlap of loci identified in either GWAS or MTAG. Panel **c** is a forest plot highlighting loci that were novel in CC-GWAS (ie, genome-wide significant in CC-GWAS but not genome-wide significant in published DCM GWAS or HCM GWAS). Association statistics for lead variants from each locus are plotted, with association results within HCM GWAS on the left (green) and DCM GWAS on the right (purple); dots and error bars represent effect sizes with 95% confidence intervals. Loci are annotated to genes using contemporary locus-to-gene mapping (Methods). Panel **d** shows results from a functional enrichment analysis^91^ testing for enrichment of all genes identified in CC-GWAS. Each dot represents a single gene set, with the x-axis showing the odds ratio for enrichment, and the y-axis showing the −log10 of the multiple-testing-adjusted *P*-value for enrichment. The dotted line represents the significance cutoff. Panel **e** is a forest plot for loci that were entirely novel in our case-case approaches (e.g., significant in either CC-GWAS or CC-MTAG but not genome-wide significant in published DCM GWAS, DCM MTAG, HCM GWAS, or HCM MTAG). Association statistics for lead variants from each locus are plotted, with association results within HCM GWAS on the left (green) and DCM GWAS on the right (purple); dots and error bars represent effect sizes with 95% confidence intervals. Loci are annotated to genes using contemporary locus-to-gene mapping (Methods). Panel **f** shows results from functional enrichment analysis for genes mapped from novel loci (e.g., loci not identified in Jurgens et al. or Tadros et al.).

Given the pervasive inverse genetic correlation between DCM and HCM, we conceptualized both disorders as phenotypic extremes along a genetic spectrum. To this end, we applied case–case GWAS (CC-GWAS)^24^, a method designed to detect variants with opposing effects between two conditions. CC-GWAS calculates case–case effect sizes from effect estimates from the respective case–control GWASs, using weights that account for sample size, sample overlap, and the expected variance–covariance structure of the effect estimates (Figure 3 and Figure 4a; Methods).

**Extended Data Figure 2:**
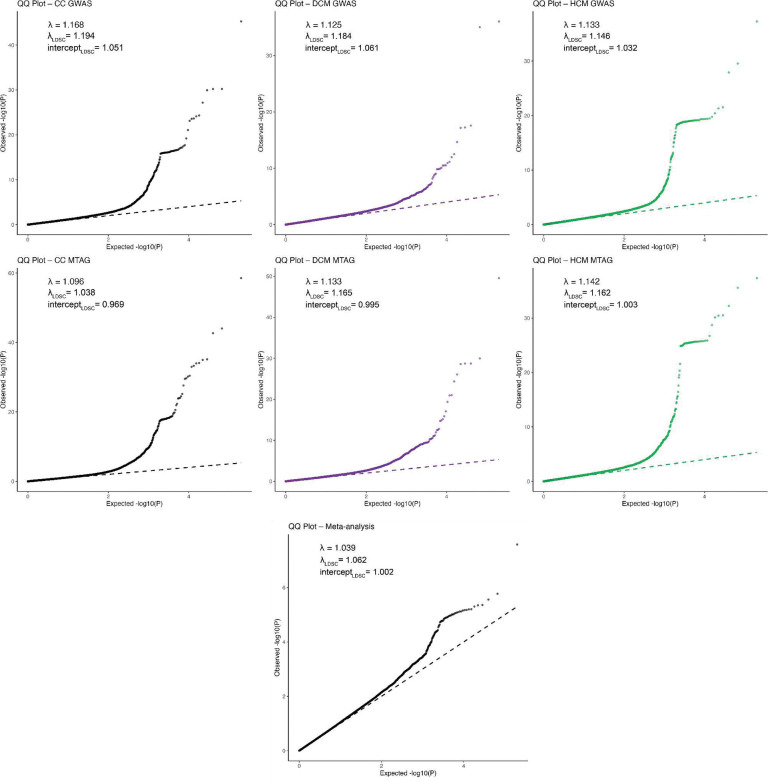
Quantile-quantile plots of contributing studies to the meta-analyses and MTAG of DCM, HCM, and CC, and shared meta-analysis. Each panel shows the quantile-quantile plot for a given GWAS, MTAG, or shared meta-analysis in a given dataset. In each quantile-quantile plot, the x-axis represents the expected −log10 of the P-value of variants under the null hypothesis, while the y-axis represents the observed −log10 of the P-value in the association study. The dashed line shows the expected calibration under the null hypothesis. For each QQ-plot, we report: i) the inflation factor λ, computed as the observed median χ^2^ statistic over the expected under the null, using all plotted variants; ii) the LDSC-based inflation factor (λ_LDSC_), which restricts to high-confidence, common variants from LD Score Regression reference panels; iii) the intercept_LDSC_, which quantifies residual inflation due to confounding (e.g., population structure, relatedness), estimated as the intercept from regressing χ^2^ statistics on LD scores.^22^ Variants are filtered to those that passed filters for inclusion into the overall analyses (Methods). P-values are derived from various logistic regression models; reported P-values are two-sided and unadjusted for multiple testing. Note: GWAS, genome-wide association study; DCM, dilated cardiomyopathy in a biobank dataset; HCM, hypertrophic cardiomyopathy; CC, case-case study.

**Extended Data Figure 3:**
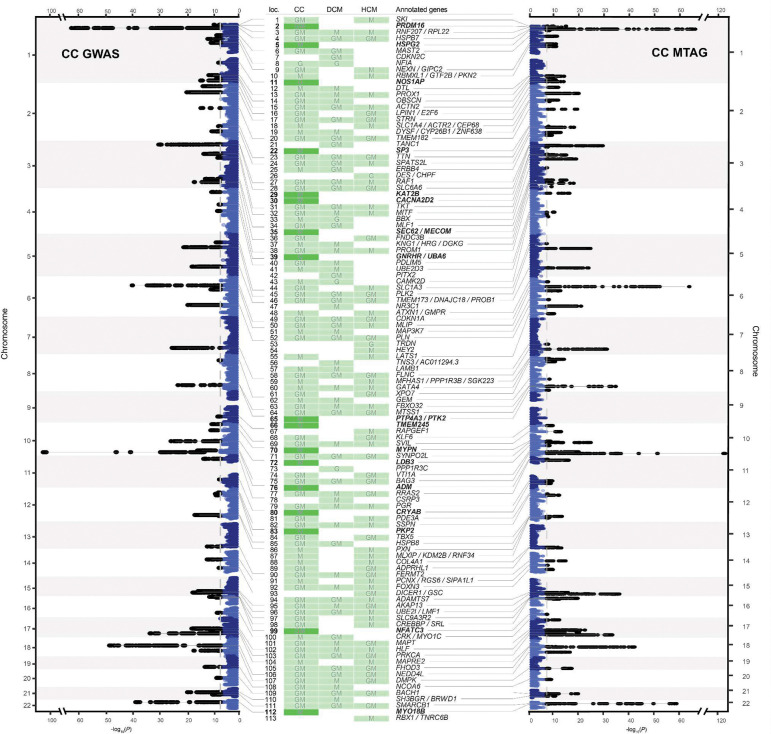
Case-case approaches identify novel loci and genes underlying the cardiomyopathy spectrum. The outer left and right parts of the figure show rotated Manhattan plots, with genomic positions on the y-axis and −log10 of the association *P*-values on the x-axis; the left Manhattan shows results from CC-GWAS, while the right shows results from an MTAG incorporating CC-GWAS with relevant cardiac MRI traits (CC-MTAG). The middle of the figure shows a heatmap for all independent loci (N=113) identified across published DCM GWAS or MTAG (Jurgens et al.), published HCM GWAS or MTAG (Tadros et al.), CC-GWAS and CC-MTAG; green indicates that a given locus was identified in a given group of GWAS, including the present case-case approaches (CC), the published DCM study (DCM), or the published HCM study (HCM); ‘G’ indicates that a given locus was identified in a GWAS approach, ‘M’ suggests that a given locus was identified in an MTAG approach, and ‘GM’ suggests that a given locus was identified in both GWAS and MTAG. Loci are annotated to highly prioritized genes using contemporary gene-prioritization methods (Methods). Notably, novel loci are highlighted in dark green, and novel genes are highlighted in bold. (**Supplementary Table 2**)

CC-GWAS showed a strong polygenic signal (λ_GC,LDSC_=1.20), while retaining adequate genomic calibration (intercept_LDSC_=1.05; Extended Data Figure 2). After applying our standardized pipeline for locus identification and gene prioritization (see Methods), CC-GWAS uncovered 67 distinct loci, including 66 that surpassed the genome-wide significance threshold and one additional locus identified through fine-mapping (rs16823802 mapping to *PRDM16*; refs.^25,26^). CC-GWAS notably enabled the discovery of 26 loci not present in either case-control GWAS dataset (Figure 3; Extended Data Figure 3, **Supplementary Table 6**), indicating a substantial boost in discovery yield using the case-case approach.

**Extended Data Figure 4:**
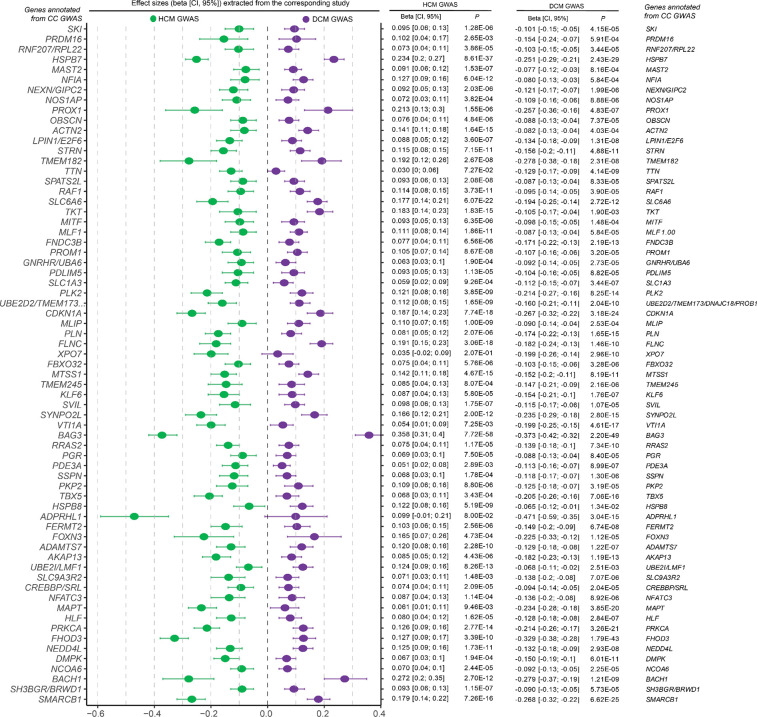
Forest plot of lead SNPs from CC GWAS loci Lead variants from each locus from CC GWAS are plotted using association results from HCM GWAS on the left (green) and DCM GWAS on the right (purple); dots and error bars represent effect sizes with 95% confidence intervals. Loci are annotated to genes using contemporary locus-to-gene mapping (**Supplementary Table 6**)

As expected, the lead variants from CC-GWAS loci showed strong patterns of inverse effects between HCM and DCM based on the input case-control data (Figure 4c; Extended Data Figure 4). For instance, when inspecting the 26 loci ‘specific’ to CC-GWAS, we found that all showed suggestive evidence of association with HCM (26/26 with *P*<0.0019=0.05/26 in HCM GWAS), and most of them showed suggestive evidence of association with DCM (24/26 with *P*<0.0019 in DCM GWAS), all with opposing directions of effect (Figure 4c). These findings support that the case-case design increased discovery power for variants with truly diverging phenotypic effects (Figure 4c).

We then assessed the genes prioritized from CC-GWAS, and found that the 26 additional loci mapped to highly relevant gene targets (Figure 4c). These include *NEXN* - encoding an actin-filament-binding protein involved in cardiac contraction^27^, *OBSCN* - encoding a large sarcomeric signalling protein involved in Ca-handling^28,29^, and *FBXO32* - encoding a ubiquitin-ligase involved in cardiomyocyte hypertrophy regulation^30^. All three genes have been linked to cardiomyopathy in humans through rare-variant associations^31–33^. Furthermore, the GWAS loci mapping to these genes were previously identified using MTAG approaches^15,16^ (Methods), indicating a degree of concordance between previous MTAG and our CC-GWAS. Nevertheless, we note that CC-GWAS identified seven loci not identified in previous MTAG, including loci mapping to *PKP2* - a major Mendelian gene for arrhythmogenic cardiomyopathy that encodes a desmosomal protein^34^, *NOS1AP* - encoding a nitric oxide synthase^35^ adaptor protein, *PRDM16* - encoding a transcription factor putatively implicated in Mendelian DCM^25,26^, and *NFATC3* - encoding a transcription factor involved in calcineurin-induced hypertrophy signaling^36^.

### Biological pathways underlying the cardiomyopathy spectrum

**Extended Data Figure 5:**
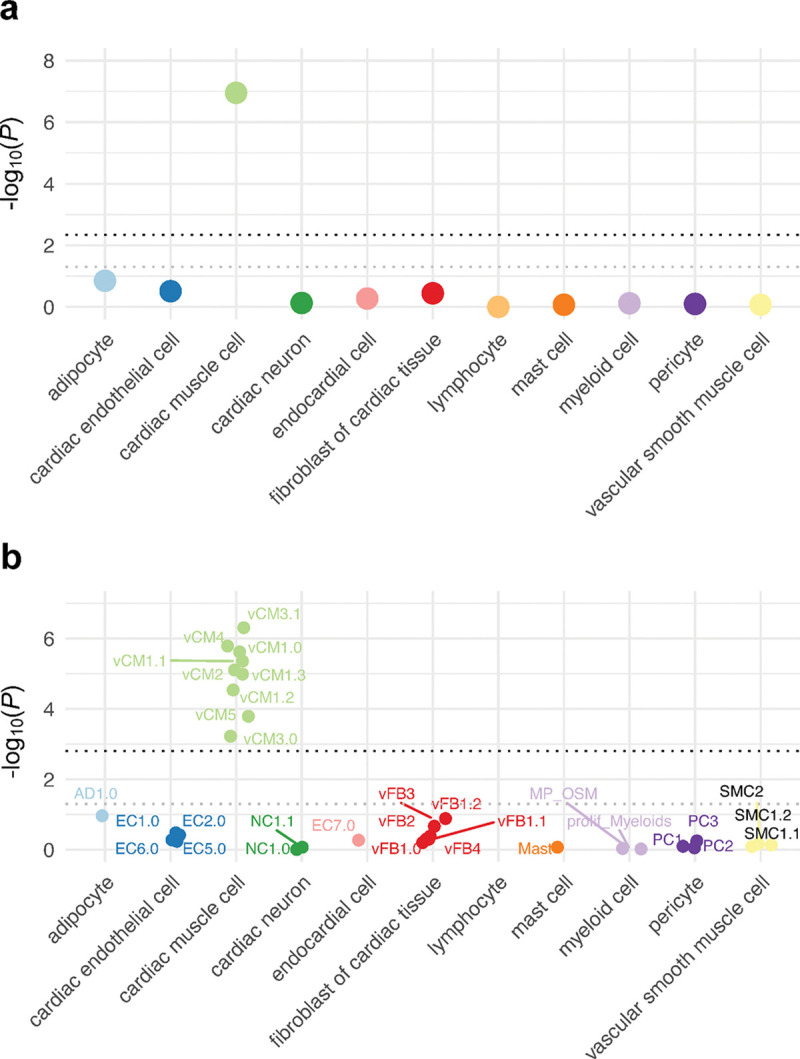
Cell type enrichment highlights a specific enrichment of cardiomyocytes in cardiomyopathy spectrum heritability. Results are derived from a cell-type-specific enrichment pipeline, modified from *sc-linker*^39^, that tests for enrichment of gene programs derived from single-nucleus RNA-seq of the the left ventricle^38^ (N=18 healthy donors). Panel **a** shows results in the form of a dot plot for curated cell types (**Supplementary Note**), while panel **b** shows results for cell states. In both panels, the x-axis represents different cell types. In contrast, the y-axis represents the −log10 of the enrichment *P*-value (enrichment based on the *Tau* statistic conditional on the baseline model). In both panels, the black dotted line represents the Bonferroni-corrected significance cutoff, while the gray dotted line represents *P*=0.05. In panel **b**, the cell states are colored by the respective cell type to which they belong. Only cardiomyocyte cell types and cell states are enriched for heritability in our CC-GWAS data.

To scrutinize the biological correlates of our CC-GWAS, we conducted analyses focused on tissue and cell-type enrichment and on genetic correlations with cardiac endophenotypes. As expected, tissue-level analyses of transcriptomic profiles^37^ revealed significant enrichment of CC-GWAS in heart and muscle tissues (**Supplementary Figure 1**). In line with previous findings for DCM^15,16^, cell type analyses using snRNAseq data^38,39^ identified strong enrichments of CC-GWAS heritability in cardiomyocytes and cardiomyocyte cell states, with no signal for other cardiac cell types (Extended Data Figure 5). Finally, we assessed the genetic correlation between CC-GWAS and endophenotypes from cardiac MRI^16^. CC-GWAS was strongly genetically correlated to main endophenotypes - including negative global circumferential strain (-Ecc) (*r*_g,global_=−0.71, *P*=6.04×10^−48^), LVESVi (*r*_g,global_=0.62, *P*=8.51×10^−40^) and LV concentricity (LVconc) (*r*_g,global_=−0.57, 4.79×10^−38^) - with *r*_g,global_ values for contractility estimated as nominally higher than for either of the case-control GWAS (Figure 2a, **Supplementary Figure 2**; **Supplementary Table 7**).

**Extended Data Figure 6:**
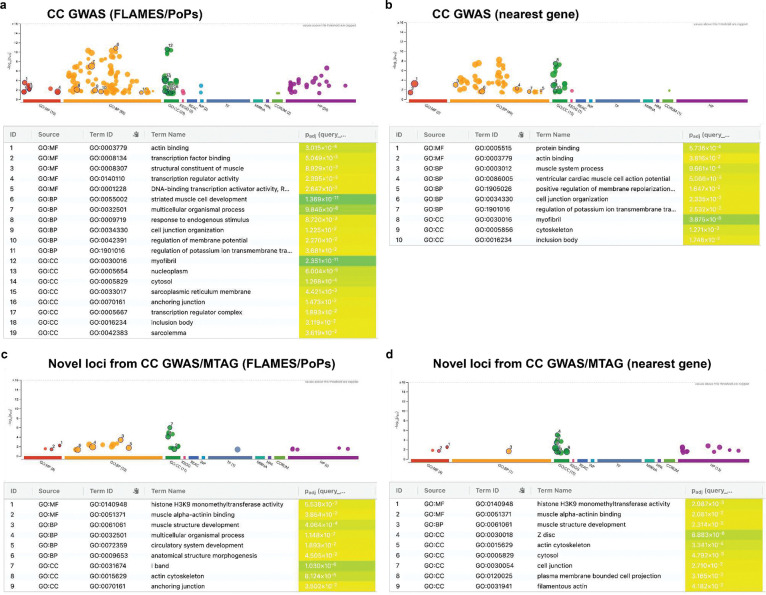
Pathway enrichment analysis of prioritized genes from case-case studies (CC GWAS/CC MTAG) using g:Profiler Pathway enrichment analysis using g:Profiler on genes prioritized from CC GWAS and CC MTAG loci. We considered 68 independent loci for CC GWAS and 17 novel loci identified in either CC GWAS or CC MTAG that were not reported in DCM or HCM GWAS/MTAG. If a locus was annotated with multiple genes (e.g., through different prioritization methods), all nominated genes were retained for downstream enrichment analysis. (**Supplementary Table 8,10**) Gene prioritization was performed using two complementary approaches: (1) FLAMES/PoPS-based prioritization, which integrates diverse functional annotations and similarity scores, and (2) a nearest-gene approach, where the gene closest to the lead SNP was selected. Panel **a** includes 77 prioritized genes from 68 CC GWAS loci (FLAMES/PoPS-based; query length = 77), and panel **b** consists of 68 genes based on nearest gene annotation (query length = 68). Panel **c** represents the 20 genes from the 17 novel loci using FLAMES/PoPS prioritization (query length = 20), and panel **d** shows the corresponding nearest gene set (query length = 18). Enrichment was tested against g:Profiler’s default gene set libraries using the hypergeometric test and Benjamini–Hochberg FDR correction. Only pathways with adjusted P < 0.05 were retained. Note: adj, adjusted; GO:MF, Gene Ontology Molecular Function; GO:BP, Biological Process; GO:CC, Cellular Component; KEGG, Kyoto Encyclopedia of Genes and Genomes; REAC, Reactome; WP, WikiPathways; TF, transcription factor targets; MIRNA, experimentally validated miRNA targets; HPA, Human Protein Atlas; CORUM, mammalian protein complexes; HP, Human Phenotype Ontology; CC GWAS, case-case genome-wide association study; CC MTAG, case-case multi-trait analysis.

To identify the biological pathways underlying the cardiomyopathy spectrum, we performed functional enrichment analysis^40^ on the genes prioritized from CC-GWAS. This analysis revealed significant enrichments for a wide range of intrinsic myocardial biological processes and molecular pathways (Figure 4d). The most significantly enriched gene sets included cardiac and muscle development (e.g., striated muscle cell development, sarcomere organization, contractile muscle fiber formation, and muscle structure development), cardiac pathophysiology (e.g., left ventricular systolic dysfunction, arrhythmia), and cytoskeletal pathways (e.g., actin cytoskeleton organization). The strong pattern of enriched cardiac-specific pathways was also obtained in sensitivity analyses using an alternative gene prioritization scheme (Methods; Extended Data Figure 6a–b; **Supplementary Table 8**). Taken together, these results indicate that molecular pathways intrinsic to cardiomyocyte homeostasis and function show pervasive inverse effects between DCM and HCM.

### Multi-trait modeling for novel locus discovery

Given the strong genetic correlation between CC-GWAS and cardiac endophenotypes, we then proceeded with an MTAG framework^41^, following a similar approach previously applied to DCM and HCM GWAS^15,16^. Notably, we integrated CC-GWAS with GWAS data for highly correlated cardiac MRI traits - including negative global circumferential strain (-Ecc), LVESVi, and LV concentricity^16^ (Methods) - from here ‘CC-MTAG’.

Genome-wide test statistics from CC-MTAG were well-calibrated (λ_GC,LDSC_=1.096; intercept_LDSC_=0.969; Extended Data Figure 2). The maximum false-discovery-rate (maxFDR) of CC-MTAG was estimated at 8.8×10^−4^, indicating a very low number of false-positives even under unfavorable circumstances^41^. Notably, the maxFDR was over 30-fold smaller than values from published MTAG analyses for DCM (0.027–0.03; refs.^15,17^) and HCM (0.03; refs.^16^). These results highlight the statistical robustness of CC-MTAG, and suggest a substantially improved locus certainty as compared to previous MTAG.

After applying our harmonized locus-mapping pipeline, CC-MTAG revealed 95 loci (**Supplementary Table 2**; Extended Data Figure 3), as compared to 63 loci in DCM MTAG and 67 loci in HCM MTAG (**Supplementary Figure 3**). Of the significant CC-MTAG loci, 94 reached genome-wide significance, and one additional locus was identified through fine-mapping (rs16823802 mapping to *PRDM16*; refs.^25,26^). Notably, 15 of these loci did not harbor any variants surpassing genome-wide significance (*P*<5×10^−8^) in either DCM or HCM MTAG^15,16^. When combining results across CC-GWAS and CC-MTAG, we identified 100 distinct loci for the cardiomyopathy spectrum (Figure 4b; Extended Data Figure 3). Partitioned heritability analyses indicated that the regions around these 100 risk loci account for up to 34.6% (SE=3.3%) of DCM and 53.4% (SE=9.6%) of HCM common-variant heritability (**Supplementary Table 9, Supplementary Note**).

**Extended Data Figure 7.**
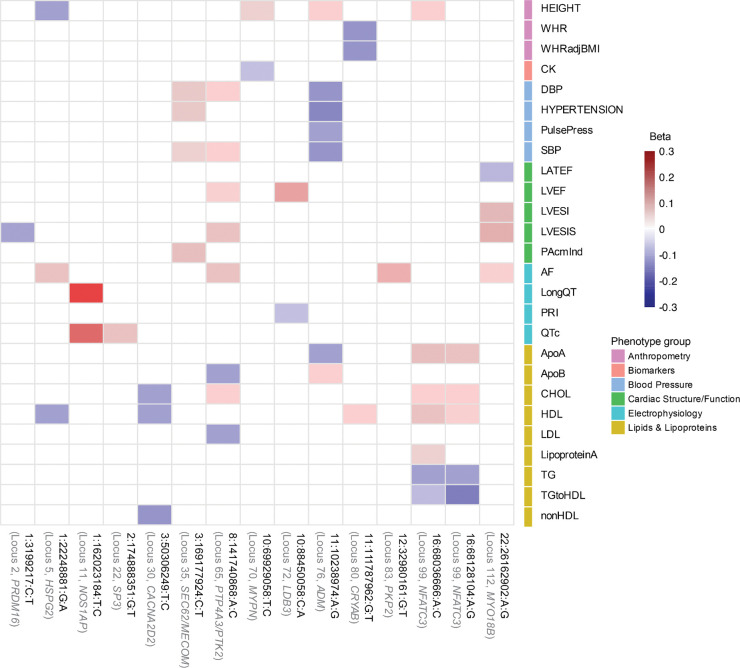
Phenome-wide association heatmap with statistically significant associations for top variants of novel loci from CC GWAS/CC MTAG Heatmap showing standardized effect sizes (β) for lead genome-wide significant variants from PheWAS using data from the Cardiovascular Disease Knowledge Portal, specifically showing associations with cardiovascular-related traits that reached the threshold of statistical significance (*P*< 5.00e^−8^). On the y-axis, each column corresponds to a lead variant, annotated with its locus ID (in the format Chromosome:Position (hg19 (GRCh37) genome build):Reference allele:Alternate allele), locus number (**Supplementary Table 11, Supplementary figure 10**), and gene. Each row represents a phenotype grouped by trait clusters (color-coded). Cells are colored by the direction and magnitude of the effect (red = positive, blue = negative), with white indicating no significant association. Only associations with non-missing β estimates are displayed. Note: AF, Atrial fibrillation; ApoA, Serum ApoA; ApoB, Serum ApoB; CHOL, Total cholesterol; CK, Creatine kinase; DBP, Diastolic blood pressure; HDL, HDL cholesterol; HEIGHT, Height; HYPERTENSION, Hypertension; LATEF, Left atrial total emptying fraction; LDL, LDL cholesterol; LVEF, Left ventricular ejection fraction; LVESI, LV end systole inferior wall thickness; LVESIS, LV end systole inferoseptal wall thickness; LipoproteinA, Lipoprotein(a); LongQT, Long QT syndrome; PAcmInd, Short-axis pulmonary artery (cm), BSA indexed; PRI, PR interval; PulsePress, Pulse pressure; QTc, Corrected QT interval; SBP, Systolic blood pressure; TG, Triglycerides; TGtoHDL, Triglyceride-to-HDL ratio; WHR, Waist-hip ratio; WHRadjBMI, Waist-hip ratio adjusted for BMI; nonHDL, Non-HDL cholesterol.

Seventeen loci, which were not identified in any of the input DCM and HCM GWAS/MTAG analyses, were considered novel. (**Supplementary Table 2**, Figure 4a–b,e). The prioritized genes mapped from the novel loci showed strong enrichments for relevant cardiac pathways (Figure 4f; **Supplementary Note**; **Supplementary Table 10**, Extended Data Figure 6b,d), and many of these loci showed relevant pleiotropic effects (Extended Data Figure 7, **Supplementary Figures 4a-s, Supplementary Table 11**). Indeed, when assessing novel loci, we found that many mapped to plausible genes (Figure 4e). These include *CRYAB*, *LDB3,* and *MYPN*, all of which have been linked to heritable cardiomyopathies through rare variation^42–45^. Other notable genes include *CACNA2D2* - encoding a voltage-gated calcium channel linked to cardiac abnormalities and bradycardia in mice^46^, *ADM* - encoding adrenomedullin, a vasodilatory peptide hormone that is currently a target of therapeutic development for heart failure^47,48^, *KAT2B* - encoding an epigenetic modifier implicated in heart development in zebrafish^49^, and *HSPG2* - encoding perlecan, a proteoglycan involved in metabolic and structural maturation of cardiomyocytes^50^.

### Independent replication of novel case-case loci

To further validate our novel loci, we then performed replication analyses in independent samples from the SHaRe (N=1,158 HCM) and GoDCM (N=1,525 DCM) cohorts (λ_GC,LDSC_=1.025; intercept_LDSC_=0.97; **Supplementary figures 9–10**), **Supplementary Note**). The genetic correlation between discovery and replication was approximately 1 (*r*_g,global_=1.06, SE=0.12, *P*=8.3×10^−19^; bivariate intercept_LDSC_=0.029). Among the 17 novel loci identified through CC-GWAS and CC-MTAG, spanning 18 lead variants, 17 variants were testable in the replication data. Of these, 16 variants showed concordant directions of effect (94.1%), a rate significantly higher than expected by chance (binomial test with null hypothesis of 50%: *P*=1.4×10^−4^). Notably, 11 variants (64.7%) replicated at nominal significance (one-sided *P*<0.05), significantly more than expected by chance (binomial test with null hypothesis of 5%: *P*=4.6×10^−11^). Effect estimates were also strongly correlated between discovery and replication (*r*=0.83, *P*=3.3e-5; **Supplementary Note, Supplementary figures 7–8**), **Supplementary table 12**).

### Drug target discovery

**Extended Data Figure 8:**
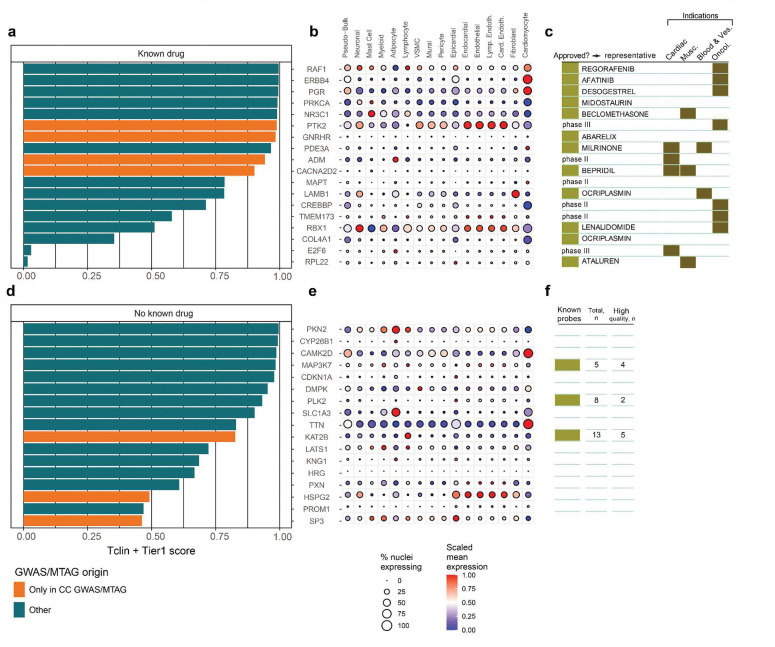
Druggability of prioritized genes **a, d.** DrugnomeAI-derived druggability scores (Tclin + Tier1 probability) for prioritized genes with high translational potential (Methods), stratified by the presence (**a**) or absence (**d**) of a known pharmacological agent in the Open Targets Platform. Orange bars denote genes identified exclusively through CC-GWAS or MTAG analyses. The x-axis reflects the Tclin + Tier1 probability, a composite score estimating the likelihood that a gene is targetable based on existing clinical and regulatory precedent. **b, e.** Single-nucleus RNA-sequencing expression profiles^38^ for the same gene sets. Dot size represents the percentage of nuclei expressing the gene in a given cell type, while color indicates scaled mean expression. **Supplementary Table 23** and **Supplementary Figure 10**. **c.** Drug development landscape for prioritized genes with known pharmacological agents. The first column indicates whether at least one drug targeting the gene is approved, with a representative agent listed. Subsequent columns summarize clinical indications for all drugs targeting each gene, categorized into cardiac diseases, muscle disorders, vascular/blood disorders, and cancer (**Supplementary Table 13**). If no drugs have reached approval, the most advanced clinical trial phase is reported. For example, *PDE3A* is the target of milrinone, an approved inotrope for acute heart failure, while *ADM* (Adrenomedullin) is currently under investigation in Phase II trials for sepsis and cardiovascular dysfunction. **f.** Availability of chemical probes for prioritized genes without known therapeutic agents. Data were obtained from the Open Targets Platform. Bars summarize the presence of known probes (left), the total number of probes (middle), and the number of high-quality probes (right) per gene. (**Supplementary Table 13**) Note: Tclin: Target of a clinically investigated drug; Tier1: Target of an FDA-approved drug; Tclin + Tier1: Combined probability score from DrugnomeAI representing gene-level druggability based on clinical and regulatory precedence; CC-GWAS: Case-case genome-wide association study; MTAG: Multi-trait analysis of GWAS; snRNA-seq: Single-nucleus RNA sequencing.

To assess the therapeutic potential of our prioritized genes, we performed a comprehensive druggability annotation by integrating tractability profiles from the Open Targets Platform (queried April 2025)^51^ with quantitative predictions from DrugnomeAI^52^ (Methods). Among the 146 prioritized genes across 113 loci identified across all DCM/HCM/CC GWAS and MTAG analyses, 12 (8.2%) were classified as “Very High tractability” due to existing approved drugs, and an additional 6 (4.1%) had “High tractability” based on late-stage clinical development (**Supplementary Table 13**). Prioritized druggable genes span established targets like *PDE3A*^53^ (the target of milrinone) and *ADM^54^*, emerging candidates such as *PGR^55^*, and novel, ligandable candidates including *MAP3K7* (*TAK1*)^56^, *PLK2*, and *KAT2B*, which collectively represent therapeutic opportunities across cardiovascular, endocrine, and stress-response pathways Extended Data Figure 8, **Supplementary Note**)^56^.

### Polygenic prediction to discriminate DCM from HCM patients

**Figure 5.**
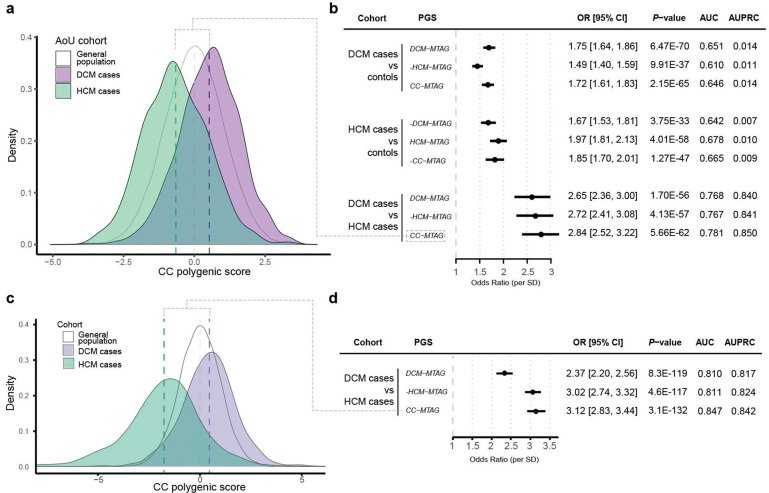
A polygenic score from case-case data strongly discriminates between DCM and HCM patients in external datasets. Panel **a** is a density plot showing the distribution of a polygenic score - built from CC-MTAG data - among independent European-ancestry samples from the *All of Us Research Program.* The x-axis represents polygenic score values, while the y-axis shows density (reflecting the relative frequency of specific polygenic score values in a certain group). The green area represents the distribution among individuals with HCM, the purple area the distribution among individuals with DCM, and the colorless area represents the distribution in other individuals (those without DCM or HCM). The green and purple dotted lines represent the means of the polygenic score values for HCM and DCM patients, respectively. Panel **b** is a forest plot showing association and prediction statistics for various polygenic scores - including one built from DCM-MTAG, one built from HCM-MTAG, and one built from CC-MTAG - across various phenotypic comparisons. The top shows comparisons where the polygenic scores are used to discriminate DCM patients from controls, the middle part shows discrimination of HCM patients from controls, and the bottom shows discrimination of DCM patients versus HCM patients. In the forest plots, some polygenic score values have intentionally been inverted to allow better comparison across different scores. Dots and error bars represent odds ratios per standard deviation of the score, with 95% confidence intervals. Odds ratios (OR) per standard deviation increase in PGS and associated P-values were obtained from logistic regression models adjusted for age, sex, and principal components 1:20. Area under the curve (AUC) and area under the precision–recall curve (AUPRC) were adjusted for principal components 1:20 only. (**Supplementary Tables 14–15**) Negative values of DCM-MTAG, HCM-MTAG, or CC-MTAG PGS were inverted (–DCM-MTAG, –HCM-MTAG, –CC-MTAG) to facilitate interpretation of effect direction and consistency. Panels **c** and **d** represent density plots and forest plots, respectively, from similar analyses within the replication cohort representing 1,158 HCM patients from SHaRe, 1,525 DCM patients from GoDCM, as well as controls from the UK Biobank.

Next, we constructed genome-wide polygenic scores^57^ from DCM MTAG (PGS_DCM_), HCM MTAG (PGS_HCM_), and CC-MTAG summary statistics (PGS_CC_), and tested these in independent samples from the All of Us Research Program^58^ (N=1,053 DCM cases, N=562 HCM cases, and N=145,976 controls; Methods). PGS_DCM_ showed the best performance in distinguishing DCM cases from controls (OR/SD=1.70 [1.61–1.81], AUC=0.651 [0.634–0.668], AUPRC=0.014), while PGS_HCM_ performed best for HCM case–control discrimination (OR/SD=1.90 [1.75–2.05], AUC=0.678 [0.655–0.701], AUPRC=0.010). Notably, however, PGS_CC_ outperformed both trait-specific scores in distinguishing DCM cases from HCM cases, and showed remarkable prediction accuracy for this task (OR/SD=2.71 [2.42–3.06], AUC=0.781 [0.758–0.805], AUPRC=0.850) (Figure 5a; **Supplementary Tables 14–15**).

To validate these findings, we also assessed the PGSs in the replication cohort comprising 1,158 HCM cases (from SHaRe) and 1,525 DCM cases (from GoDCM). PGS_CC_ again provided the strongest discrimination between DCM and HCM cases, with an OR/SD of 3.12 (95% CI 2.83–3.44; *P*=3.1×10–132), and the highest predictive performance (AUC=0.85, AUPRC=0.84; Figure 5b, **Supplementary table 16, Supplementary Note**). These findings further validate our case-case GWAS approach and highlight the potential of PGS to place individuals on the genetic cardiomyopathy spectrum.

### Leveraging shared genetics for discovery

**Extended Data Figure 9:**
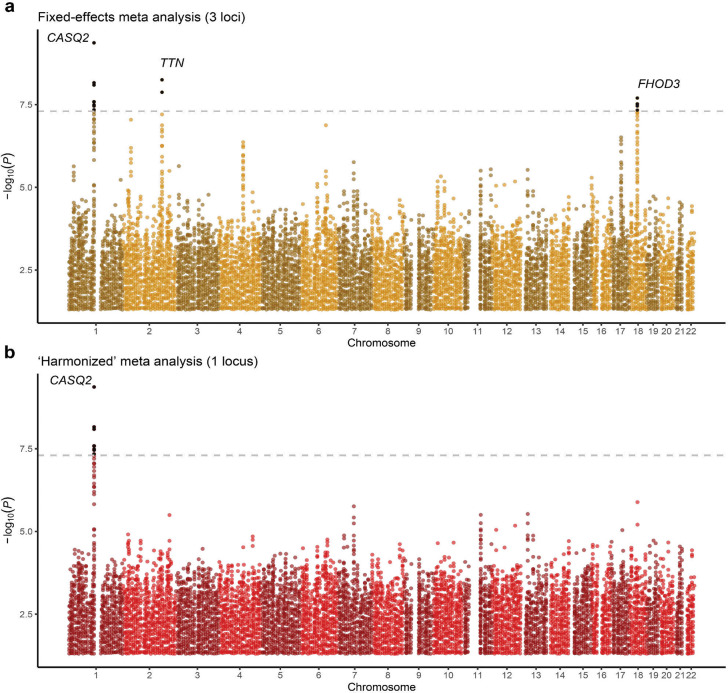
Manhattan plots for two stages of shared effect meta-analysis Both panels are Manhattan plots where each dot represents a genetic variant, with genomic positions on the x-axis and −log10 of the association P-value on the y-axis. Panels **a** shows results for meta-analysis statistics using a regular fixed-effects inverse-variance-weighted approach (3 loci). Panel **b** shows results after a second-stage meta-analysis under a random-effects framework (1 locus) (Methods)

To directly contrast our case-case approaches, we then performed a ‘shared-effect’ meta-analysis in which we treated DCM and HCM as similar diseases (Methods). This approach showed a substantial depletion of genome-wide significant loci, as compared to the input case-control GWAS: We identified only three significant loci using an initial fixed-effects meta-analysis, with only one significant locus - which was novel - remaining after applying a harmonized random-effects meta-analysis (Figure 3d; Extended Data Figure 9). These findings are consistent with a substantial portion of bona fide cardiomyopathy risk loci being canceled out in this meta-analysis due to opposing effects.

**Figure 6:**
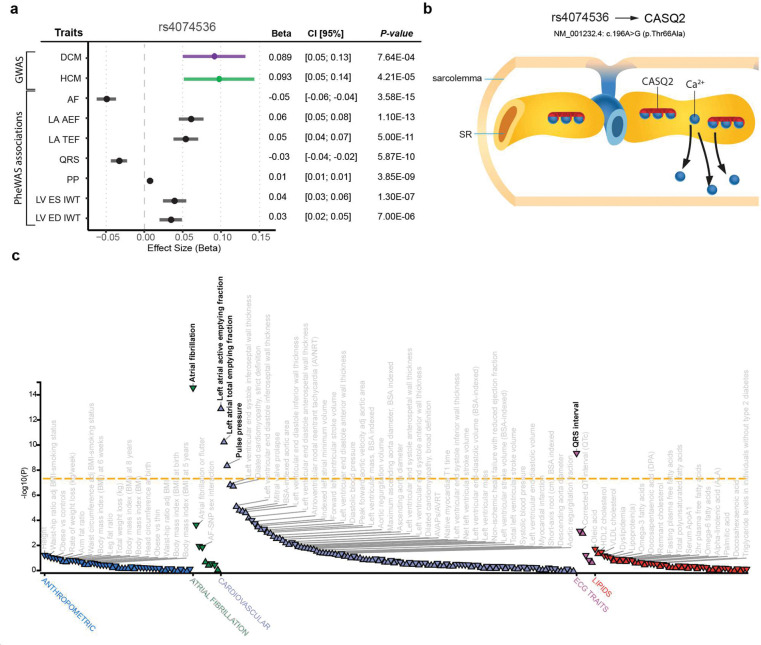
A shared effects meta-analysis identifies calcium handling as a potentially concordant mechanism underlying DCM and HCM. Panel **a** is a forest plot for the lead variant (rs4074536) identified in the shared-effect meta-analysis in which DCM and HCM were modeled as similar diseases; the forest plot shows the association statistics for rs4074536 in DCM GWAS (purple), HCM GWAS (green), as well as significant findings from a phenome-wide association (PheWAS) look-up in the Cardiovascular Disease Knowledge Portal and OpenTargets. Dots and error bars represent beta coefficients and 95% confidence intervals. Panel **b** is a schematic showing the potential effect of the rs4074536 on *CASQ2* function and calcium handling. Rs4074536 causes a missense change (p.Thr66Alu) in *CASQ2*, encoding a calcium-binding protein in the sarcoplasmic reticulum of the cardiomyocyte^92^. Panel **c** shows broader results from PheWAS using data from the Cardiovascular Disease Knowledge Portal, specifically showing results from cardiovascular-related traits. The x-axis represents different traits grouped by trait clusters, while the y-axis represents the −log10 *P*-value for the association between rs4074536 and the respective traits.

The single novel locus showed similar effect estimates for DCM and HCM (Figure 6a) and was plausibly related to cardiac pathology. Notably, the lead variant was a missense variant (rs4074536; p.Thr66Ala) in *CASQ2*, a calcium-binding protein in the sarcoplasmic reticulum (SR) of myocytes that regulates Ca-release and excitation^59^ (Figure 6b). Rare *CASQ2* loss-of-function variants are known to underlie catecholaminergic polymorphic ventricular tachycardia, a lethal arrhythmic syndrome with structurally normal hearts^60^. Nevertheless, in phenome-wide association analyses, besides association with a shorter QRS duration and decreased risk of atrial fibrillation, p.Thr66Ala was associated with increased LV wall thickness and increased atrial contractility, suggesting a structural phenotype (Figure 6a,c, **Supplementary Table 13**). These results indicate that distinct molecular pathways related to calcium-handling might be concordant between DCM and HCM.

To replicate this novel locus, we compared allele and genotype frequencies of the lead variant in replication cohorts (SHaRe and GoDCM) to non-Finnish European controls from gnomAD (v4.1), observing significant enrichment of the effect allele in both HCM (OR=1.14, one-sided *P*=0.002) and DCM (OR = 1.07, one-sided *P*=0.037) (**Supplementary table 18, Supplementary Note**).

### Concordance of cardiometabolic pathways for DCM and HCM

**Figure 7.**
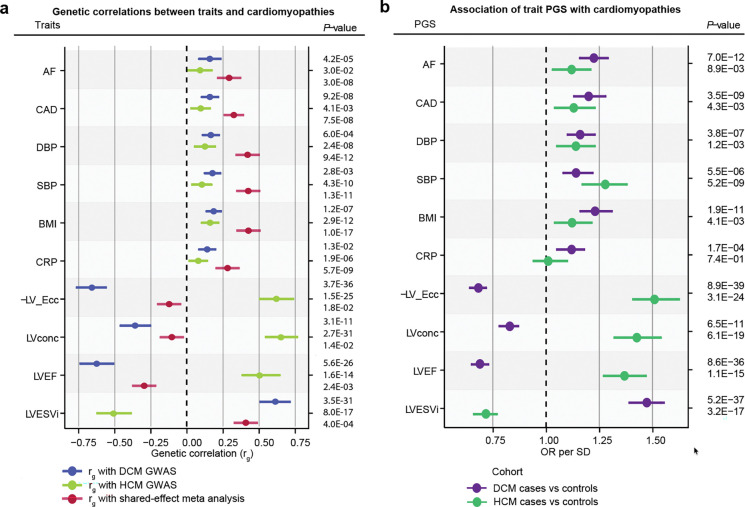
Cardiometabolic and cardiac MRI traits reveal shared and opposing genetic architecture across cardiomyopathies Panel a is a forest plot showing genetic correlations (r_g_) between GWAS of cardiometabolic traits/diseases and GWAS of DCM, HCM, and shared-effect meta-analysis, estimated using bivariate LD score regression. The x-axis shows (r_g_) with 95% confidence intervals. Blue, olive, and dots indicate correlations with DCM GWAS, HCM GWAS, and the shared-effect meta-analysis, respectively. (**Supplementary Table 21)** Panel b shows associations of trait-polygenic scores (PGS) with DCM and HCM status in the All of Us Research Program (European-ancestry subset). PGSs were derived from publicly available GWAS for cardiometabolic traits (AF^64,67^, CAD^68^, BMI^69^, SBP^67,70^, DBP^71^, CRP^72^) and cardiac imaging traits (LVconc, –Ecc, LVEF, LVESVi)^16^. The x-axis represents odds ratios (OR) per standard deviation of each PGS, with 95% confidence intervals. Purple and green dots represent associations with DCM vs controls and HCM vs controls, respectively. (**Supplementary Table 22)** *P*-values for both PGS associations and genetic correlations are shown on the right side of each panel.

Despite uncovering only one genome-wide significant locus, the shared-effect meta-analysis revealed strong positive genetic correlations with various HF outcomes, as well as with various cardiometabolic traits^61,62^ and diseases^63–65^ (**Supplementary Note**). The strongest associations were observed with non-ischemic heart failure (niHF)^63^ (*r*_*g*_=0.69, *P*=3.05×10^−19^), all-cause HF^63^ (*r*_*g*_=0.677, *P*=7.87×10^−34^), diastolic and systolic blood pressure (DBP: *r*_*g*_=0.415, *P*=5.00×10^−5^, SBP: *r*_*g*_=0.375, *P*=1.50×10^−4^), body mass index (BMI) (*r*_*g*_=0.407, *P*=1.00×10^−4^), body weight (*r*_*g*_=0.392, *P*=1.50×10^−4^) and C-reactive protein (CRP) (*r*_*g*_=0.268, *P*=6.00×10^−4^). Associations with 21 other traits and disease endpoints were nominally significant (*P*<0.05). (Figure 7a; **Supplementary figure 9, Supplementary Tables 19, Supplementary Note**). In contrast, the genetic correlations between these cardiometabolic traits with DCM and HCM were, in general, substantially smaller.

To validate these findings, we constructed polygenic scores (PGS)^66^ from publicly available GWAS of select cardiometabolic traits/diseases that showed significant genetic correlation with our shared-effect meta-analysis (AF^64,67^, CAD^68^, BMI^69^, SBP^67,70^, DBP^71^, CRP^72^). We tested these for association with DCM and HCM among independent samples from the All of Us Research Program^58^. For comparison, we also created PGS for cardiac MRI-derived traits—LV concentricity (LVconc), negative circumferential strain (-Ecc), LV ejection fraction (LVEF), and indexed end-systolic volume (LVESVi)^16^. Mirroring the genetic correlations, cardiometabolic PGSs demonstrated concordant directional associations with DCM and HCM, whereas cardiac functional PGSs exhibited divergent effects (Figure 7b, **Supplementary Tables 20–22**). Therefore, despite a strong inverse genetic architecture of intrinsic myocardial function, a genetic component of cardiometabolic health may be concordant across DCM and HCM.

## Discussion

Here, we utilized large GWAS data for DCM and HCM to study the shared and opposing genetic pathways underlying these heritable cardiomyopathies. Despite being associated with similar adverse outcomes, we showed that DCM and HCM are strongly inverse on the global, regional, and local genomic levels. Leveraging these opposing effects with case–case approaches - treating DCM and HCM as opposite entities on a genetic spectrum - identified 100 distinct loci (17 novel), which strongly converged on intrinsic myocardial functional pathways. Polygenic scores derived from the case–case data showed strong predictive accuracy in two independent datasets for discriminating DCM patients from HCM patients. Nevertheless, a shared-effect GWAS identified one signal - a novel locus mapping to *CASQ2* - and highlighted concordant genetic associations with cardiometabolic risk factors and traits. These findings permit several conclusions.

First, our results demonstrate the value of statistical case-case approaches for locus and gene discovery in the context of disease spectra related to one organ. Prior case-case GWAS have mainly focused on identifying divergent loci across similar diseases with positive genetic correlation^24,73,74^. In contrast, we show that case–case approaches can also yield improved locus discovery for diseases that represent genetic opposites, as is the case for DCM and HCM across multiple genomic levels. Notably, our case-case GWAS appeared to strongly capture the genetics of intrinsic myocardial structure and function, as evidenced by enrichment in cardiomyocyte-specific cell types and states and by strong genetic correlations with cardiac MRI endophenotypes. Indeed, while prior case-case studies typically identified only a handful of significant loci^24,73,74^, our analyses provide an extensive map of common genetic variation underlying the heritable cardiomyopathy spectrum.

Second, the largely opposing pathways underlying DCM and HCM yield broad insights for therapeutic development. The vast number of loci from our case-case GWAS - and the near-absence of loci from the shared-effect analysis - underscore the pervasive inverse architecture of cardiomyocyte-intrinsic pathways in the pathogenesis of DCM and HCM. Notably, functional studies have previously established inverse genetic mechanisms for rare genetic variants, pertaining to cardiomyocyte contractility, calcium sensitivity, and myosin motor function^12–14^. This inverse pattern is illustrated clinically for cardiac myosin inhibitors used for treatment of obstructive HCM^75–77^, as a complication of these medications is systolic dysfunction^75,78^(a key feature of DCM^79^). Adding to this prior knowledge, our data support a broader inverse nature of cardiomyocyte homeostatic pathways across DCM and HCM (including proteostasis, transcriptional regulation, and intracellular signaling pathways). As such, therapeutics directly targeting the cardiomyocyte may broadly be expected to exhibit opposing utility in DCM and HCM, and may further imply that such therapeutics have narrow therapeutic windows, potentially informing future drug discovery and repurposing efforts.

Beyond broader mechanistic insights, our case-case data also provide an extensive list of prioritized genes with potential druggability data, putting forward several targets for therapeutics in the cardiomyopathy space. A notable example is *ADM*, which encodes the vasodilatory peptide adrenomedullin. Elevated adrenomedullin levels have long been observed in heart failure, and Adrecizumab—a non-neutralizing adrenomedullin-binding antibody—progressed to phase II clinical testing for acute heart failure (ClinicalTrials.gov ID: NCT04252937). However, no updates have been reported since 2021^54^.

Third, our case-case PGSs show utility for placing individuals on the genetic cardiomyopathy spectrum. These scores could potentially be applied in clinical scenarios where distinguishing cardiomyopathy subtypes is challenging—such as in cases of phenotypic convergence. A notable example is ‘burned-out’ HCM, which may resemble DCM clinically, and which may be diagnosed as DCM, yet remains genetically distinct and positioned at the opposite end of the cardiomyopathy spectrum^80^. Nevertheless, we note that such clinical scenarios are expected to be quite rare. Therefore we stress that disease subtype prediction by PGS should be considered conceptual and illustrative for the broader genetics field at this stage. Still, future work could assess how and when PGS, and potentially other clinical and genetic modalities, may be utilized for subtype discrimination in heart failure, in the future.

Fourth, despite the broadly inverse mechanisms, specific cardiomyocyte-intrinsic calcium-handling pathways may be shared between DCM and HCM. The single significant locus from our shared-effect analysis mapped to a missense variant in *CASQ2*, encoding calsequestrin, an important calcium-scavenger in the sarcoplasmic reticulum (SR) of cardiomyocytes. While calcium leak/overload in the SR has been strongly implicated in DCM and heart failure more broadly^81–83^, diastolic calcium leak has also been involved in the diastolic dysfunction associated with HCM^84^ and loss of calsequestrin has been found to increase hypertrophic signalling in murine models^85,86^. Other recent murine work, however, has suggested that altered SR function mainly affects arrhythmic susceptibility rather than primary structural remodeling in cardiomyopathy^87^. Future studies are needed to assess whether modulation of calsequestrin represents a therapeutic modality with utility across heritable cardiomyopathies.

Fifth, our data indicate that the genetic components of certain extra-cardiac risk factors are concordant in DCM and HCM. Indeed, previous work has shown that certain risk factors, such as hypertension and obesity, may be shared between DCM and HCM^15,16,88,89^. While we were not able to recapitulate loci for these risk factors in our shared-effect GWAS, presumably due to their highly polygenic nature^71,90^, genetic correlations and polygenic risk score analyses highlighted strong concordant genetic links between these risk factors and both DCM and HCM. Thus, despite opposing myocardial mechanisms, our data support similar approaches to cardiometabolic risk management in both cardiomyopathies.

In conclusion, DCM and HCM exist at opposite ends of a genetic disease spectrum, which is closely linked to intrinsic myocardial structure and function. By leveraging the opposing and shared genetic pathways underlying these heritable cardiomyopathies, we identify many loci and effector genes across this spectrum, with potential implications for therapeutic development and our understanding of the the polygenic architecture of disease subtypes.

## Online Methods

### Ethics

The main discovery analyses, based only on statistical analysis of summary statistics, were covered under a study protocol approved by the Medical Ethical Review Committee of the Amsterdam UMC. Use of AllofUs data was approved under a data use agreement between Amsterdam UMC and the All of Us Research Program; the NIH All of Us Research Program Institutional Review Board approved the All of Us study. For SHaRe and GoDCM, institutional review board and ethics approvals were obtained in accordance with policies applicable to each participating site. All study cohorts either collected informed consent from research participants or received appropriate approval from ethical/review committees to waive the requirement of informed consent.

### Data sources and processing

To investigate the genetic similarity between dilated cardiomyopathy (DCM) and hypertrophic cardiomyopathy (HCM), we analyzed summary statistics from two recent genome-wide association studies (GWAS).

For DCM, we used data from a large-scale meta-analysis by Jurgens et al. (2024),^15^ which included 9,365 cases and 1,199,156 controls of European ancestry, and identified 38 genome-wide significant loci (*P*<5 × 10^−8^). This study further integrated the DCM GWAS with data from MRI-derived left ventricular (LV) traits— global circumferential strain, end-systolic volume (LVESV), and ejection fraction (N = 36,083)^16^ —using multi-trait analysis of GWAS (MTAG),^41^ resulting in 65 significant loci. For HCM, we used summary statistics from Tadros et al. (2025), who analyzed 5,900 clinically diagnosed HCM cases and 68,359 controls, with 34 loci reaching genome-wide significance. Similarly, the HCM GWAS was integrated with MRI traits (global circumferential strain, LV end-systolic volume (LVESV), and LV concentricity) using MTAG, yielding 68 genome-wide significant loci. In our study, we used the single-trait GWAS and MTAG summary statistics for downstream analyses for both DCM and HCM. Further details on the studies are presented in the **Supplementary Note**.

To ensure robust variant representation, we applied study-specific filtering thresholds: for DCM summary statistics, we retained variants with ≥70% effective sample size; for HCM, we retained those with ≥96% of the total sample size. Variants within the extended *MYBPC3* region (chr11:29,978,453–80,288,956) were excluded due to potential confounding from rare founder variants (**Supplementary Note**). After filtering, 6.6M, 6.0M, 5.5M, and 5.1M variants remained for the DCM GWAS, HCM GWAS, DCM MTAG, and HCM MTAG datasets, respectively (**Supplementary Table 1**).

### Genetic correlations on global and regional levels

We aimed to assess the genetic similarity between DCM and HCM on several genomic levels. To this end, we used the GWAS summary statistics to determine the genetic similarity on the global level using linkage disequilibrium score regression (LDSC)^93^, and on the regional level using Local Analysis of [co]Variant Association (LAVA)^23^.

#### Global genetic correlation

The global genetic correlation (*r*_g,global_) represents the genetic covariance between two traits based on the cumulative effects of variants from GWAS. To estimate *r*_g,global_ between DCM and HCM, we applied bivariate LDSC (v.1.0.1)^22^ to the processed DCM and HCM GWAS summary statistics. The European subset of the 1000Genomes project (v.3.5) dataset was used as a linkage disequilibrium (LD) reference panel, and analyses were subsetted to nonambiguous HapMap3 variants. Hypothesis tests were performed using a null hypothesis of *r*_g,global_=0, using two-sided tests.

We also aimed to estimate *r*_g,global_ between DCM and HCM, conditional on other heritable traits. To this end, we computed pairwise genetic correlations between DCM, HCM, and various cardiomyopathy risk factors^15,18^, including systolic blood pressure^61^, diastolic blood pressure^61^, body-weight^61^ and body-mass-index^62^. To estimate *r*_g, global_ conditional on these risk factors, we used Genomic Structural Equation Modeling in the GenomicSEM R package (v0.0.5) (**Supplementary Note**). Similarly, we assessed the genetic correlation between DCM and HCM conditional on LV traits^16^ (global circumferential strain, LVESVi, and LV concentricity) (**Supplementary Note**).

Bivariate LDSC analyses were also performed to estimate the genetic correlation between various cardiomyopathy GWAS (eg, DCM case-control, HCM case-control, case-case-GWAS, and shared-effect GWAS; see below) against a suite of other HF outcomes, cardiometabolic traits/diseases, and cardiac MRI traits (**Supplementary Table 21**). Cardiometabolic traits were selected based on those that exhibited the strongest genetic correlation in the the shared-effect meta-analysis. Then, GWAS summary statistics for these traits were used to construct PGS. HF was excluded from this analysis due to the heterogeneity of mechanisms and the difficulty of interpretation.

#### Regional genetic correlation

In addition to global correlation analysis, we explored the correlation between DCM and HCM within smaller genomic regions. To this end, we used LAVA (v0.0.7), a tool developed to estimate heritability (*h*^2^_regional_) and genetic correlation (*r*_g,regional_) from GWAS summary statistics within genomic regions.^23^

We used 2,495 genome-partitioned LD blocks as defined by the LAVA developers^23^. Within the LAVA framework, we first conducted univariate association analyses for DCM and HCM to identify regions with significant *h*^2^_regional_ for both traits. To this end, we overlapped the LD regions with variant positions from the DCM and HCM GWAS summary statistics, yielding 2062 testable regions for DCM GWAS and 2,161 for HCM GWAS. Therefore, 4,223 univariate tests were conducted, for which the null-hypothesis represented *h*^2^_regional_=0, and for which two-sided *P*<0.05/2,495 was considered significant. Next, bivariate analysis was performed within the 101 regions that showed significant *h*^2^_regional_ for DCM and HCM. The null-hypothesis for the bivariate analysis was represented by *r*_g,regional_=0. Regions with significant *r*_g_ were subsequently identified if they reached two-sided *P*<0.05/101 in this bivariate analysis.

### Case-case genome-wide association study

To model DCM and HCM as genetically opposite, we constructed a case-case GWAS (CC-GWAS) in which DCM cases were compared to HCM cases in a GWAS framework. To construct CC-GWAS from our available GWAS summary statistics, we used the *CCGWAS* R-package (v0.1.0)^24^. *CCGWAS* estimates the genetic difference between cases of two diseases, by leveraging differences in allele frequencies using a genetic distance measure, F_ST,causal_. *CCGWAS* quantifies genetic divergence and computes case-case association statistics by leveraging the variances and covariances of effect sizes across three scenarios (**Supplementary Note**): DCM cases versus controls (DCM1/DCM0), HCM cases versus controls (HCM1/HCM0), and DCM cases versus HCM cases (DCM1/HCM1). This approach allows for the reconstruction of case-case association statistics, and therefore the detection of loci with divergent effects between two disease states.

For this analysis, we used the processed summary statistics from DCM and HCM GWAS, along with the following input parameters: i) the assumed population prevalences (0.4% for DCM, 0.2% for HCM); ii) the case/control numbers in DCM GWAS and HCM GWAS, with some attenuation for potential missingness (**Supplementary Note**); iii) the heritabilities from LDSC (14.2% for DCM, 18% for HCM); iv) the *r*_g,global_ between DCM and HCM, and its intercept, from LDSC (*r*_g,global_=−0.56 and error_covariance_=0.012)^22^; and v) the number of effectively independent causal variants for DCM (1223; **Supplementary Note**)^24^. Naturally, CC-GWAS was restricted to genetic variants identified in the processed DCM and HCM GWAS summary statistics; after additional automatic filtering by the *CCGWAS* package, 4987309 high-quality variants remained in the CC-GWAS analysis and in the resulting summary statistics. Genome-wide significance was defined as *P*<5×10−8, and all hypothesis tests were two-sided.

#### Case-case MTAG

To enhance discovery power for our case-case approach, we applied the MTAG framework (v1.0.8). As mentioned before, MTAG approaches have been successfully applied to boost genomic discovery for DCM and HCM in previous literature^15,16,18^. In short, MTAG leverages *r*_g,global_ between a target GWAS and genetically related traits to construct association statistics with higher precision, while accounting for sample overlap. In the present analysis, our target trait was the CC-GWAS. As in previous work, we selected LV traits from cardiac MRI as secondary phenotypes.

To identify relevant secondary traits and ensure trait compatibility, we first estimated pairwise *r*_g,global_ between CC-GWAS and 10 LV traits from a previous GWAS of cardiac MRI traits (n = 36,083 UK Biobank participants)^16^(**Supplementary Table 7**). The MTAG developers recommend including traits with substantial correlations (typically *r*_g, global_ > 0.65) to maximize power and reduce false positives. In our data, we identified three major clusters of genetically correlated traits (a ‘contractility’ cluster, a ‘volumetric’ cluster, and a ‘hypertrophy’ cluster; **Supplementary Figure 2)**. From these clusters, we selected three highly correlated index traits—global circumferential strain, LVESVi, and LV concentricity—with *r*_g_ values of 0.793, 0.711, and −0.668, respectively. These were included alongside CC-GWAS in the final MTAG^41^. This MTAG resulted in an estimated 102% increase in effective sample size for CC-GWAS, reflecting a substantial increase in statistical power. MTAG also estimated a maximum false discovery rate (FDR) of 0.00088, reflecting the expected proportion of spurious findings under the most conservative assumption of trait-specific effect size distributions. Genome-wide significance was defined as *P*<5×10−8, and all hypothesis tests were two-sided. Results from this analysis are hereafter referred to as ‘CC-MTAG’.

### Shared-effect genome-wide association study

We then performed a GWAS meta-analysis across DCM and HCM, assuming that both cardiomyopathies represent genetically similar diseases. First, we computed initial meta-analysis statistics using a regular fixed-effects inverse-variance-weighted approach, using the MTAG software (constraining *r*_g,global_=1). We restricted this meta-analysis to variants found at high-quality in both the DCM and HCM GWAS summary statistics. For any given variant, large differences in estimate precision may affect the meta-analysis. For example, if a variant is much better-powered in the DCM GWAS, the results will be disproportionately influenced by DCM. We therefore removed variants with outlying differences in precision (**Supplementary Note**), leaving 4,843,297 genetic variants for the shared-effect meta-analysis.

To account for the fact that DCM and HCM are likely not genetically identical, we performed a second-stage meta-analysis using a random-effects framework. The random-effects model assumes that the true effect sizes are similar, but not identical, allowing for some heterogeneity between DCM and HCM effects. We performed a random-effects inverse-variance-weighted meta-analysis using the *meta* R-package (v7.0). For any variants with *P*<1e-4 in the first stage, we used the random-effects *P*-value if it was more conservative than the fixed-effects *P*-value.

### Locus definitions, variant annotation, and gene prioritization

Locus definition, variant annotation, and gene prioritization were performed using a unified pipeline across all summary statistics, including DCM GWAS, DCM MTAG, HCM GWAS, HCM MTAG, CC-GWAS, and CC-MTAG. (Code availability)

#### Processing of summary statistics using FUMA

Each set of summary statistics was first processed using Functional Mapping and Annotation (FUMA)^37^ v1.6.1 (https://fuma.ctglab.nl/). Among other analyses, FUMA applies Multi-marker Analysis of GenoMic Annotation (MAGMA; v.1.08) to perform an initial gene-based association analysis, by aggregating variant-level signals into gene-level statistics while accounting for linkage disequilibrium^94^. The MAGMA gene-level scores were also used by FUMA to test for tissue-specific enrichment of RNA expression profiles, based on transcriptomic profiles across dozens of tissues from the GTEx v8 dataset (GTEx/v8/gtex_v8_ts_general_avg_log2TPM)^95^. The MAGMA gene scores and tissue enrichment statistics were used as input for our gene prioritization pipeline, as described in detail below.

#### Fine-mapping and credible set formatting

Our gene nomination pipeline required finding credible sets that likely include the causal variants from the respective GWAS. To identify such credible sets, we performed fine-mapping using the SuSiER algorithm (v0.12.35)^96,97^. For each GWAS dataset, the SuSiER algorithm was run within separate LD blocks derived from UK Biobank European-ancestry reference data.^98^ The minimum squared correlation was set to 0.5 (the default), unless the algorithm failed to converge, in which case we relaxed the threshold to 0.25. If SuSiE continued to fail in a region harboring genome-wide significant variants, we flagged the respective LD region and generated an artificial credible set using only the most significant variant in the region **(Supplementary Tables 25–36)**

#### Gene prioritization using FLAMES

To perform gene prioritization, we used the recently described ‘fine-mapped locus assessment model of effector genes’ (FLAMES) approach (v1.1.1)^21^. FLAMES combines two main approaches to gene prioritization in a weighted framework to compute causal gene predictions that outperform prior methods. In particular, FLAMES first uses pre-fit machine learning models (based on XGBoost) to link fine-mapped variants to likely effector genes, based on various parameters, including variant-to-gene distance, epigenomic context, and quantitative trait loci. Second, FLAMES uses the Polygenic Priority Score (PoPS^99^) method to learn gene features associated with the trait from functional networks; these features consist of cell-type-specific gene expression, biological pathways, and protein–protein interactions (PPIs). We then applied the FLAMES framework to each of our GWAS datasets. To this end, for a given GWAS dataset, we first ran PoPS (v0.2)^99^ using the MAGMA Z-scores as input and the full feature matrix provided by the PoPS developers. We then annotated each credible set using the *annotate* module from FLAMES, which combines variant-to-gene mappings, MAGMA Z-scores, PoPS scores, and GTEx tissue enrichment data.

FLAMES then returned a ranked list of genes per locus in *FLAMES_scores.preds*, including raw and scaled FLAMES scores, XG-Boost scores, PoPS scores, and estimated precision.

#### Locus definition and consolidation across studies

For each credible set, we selected the top variant based on the highest posterior inclusion probability (PIP), or, in cases where fine-mapping failed, the variant with the lowest *P*-value. All index variants were then sorted by chromosome and genomic position. Index variants located within 1Mb of one another were merged into one locus, to define non-overlapping genomic loci. Each locus was assigned a unique identifier based on its genomic position, with consistent numbering maintained across all analyses (**Supplementary Table 2**).

#### Gene prioritization across studies

Despite applying a harmonized pipeline for gene prioritization across the various GWAS datasets, it was possible for the FLAMES algorithm to nominate different causal genes within the same locus in different GWAS datasets. To consolidate gene-level evidence within and across datasets, we therefore applied a scoring framework to prioritize effector genes at each locus. For each study, genes identified as top-ranked by either PoPS or FLAMES were assigned 0.5 points per method per study. Scores were then aggregated across all studies. For example, locus 12 (chr1:212,107,306–212,277,107) appeared in both DCM MTAG and CC MTAG. In DCM MTAG, *DTL* was prioritized by both PoPS and FLAMES (score = 1), while in CC MTAG, *BATF3* was prioritized by PoPS and *DTL* again by FLAMES. This resulted in cumulative rank scores of *DTL* = 1.5 and *BATF3* = 0.5. Accordingly, *DTL* was selected as the reported gene for this locus.

For each locus, the gene(s) with the highest total score were designated as lead candidates. In cases where multiple genes had equal scores, or where the difference between top-scoring genes was <1.0, all were retained as joint candidates. This strategy enabled the identification of both study-specific and consensus lead genes across DCM, HCM, and case–case analyses. While we acknowledge that the approach is, to some extent, arbitrary, we applied it to indicate instances where gene prioritization produced potentially inconsistent results transparently. Reassuringly, we found that a single effector gene was nominated in the vast majority of loci using this approach (Extended Data Figure 3). The final locus-level summary included genomic coordinates, contributing studies, top-ranked genes, prioritization scores, and selected lead gene(s). **(Supplementary Table 2)**.

### Enrichment analyses

#### Tissue enrichment

As described, tissue-specific expression enrichment of prioritized genes was assessed using bulk RNA sequencing data from GTEx v8, as implemented in the FUMA platform^37^. These results were used to identify tissue-specific gene expression profiles that were significantly enriched for CC-GWAS genes. Statistical significance was evaluated using a two-sided hypergeometric test, corrected for multiple testing using the Benjamini–Hochberg method.

#### Cell type enrichment

To identify the cardiac cell types contributing to cardiomyopathy-spectrum heritability, we applied a cell type-specific heritability enrichment framework^39^. To this end, we used single-nucleus RNA-sequencing (snRNAseq) data from non-failing hearts from 18 donors^38^. (downloaded from https://www.ncbi.nlm.nih.gov/geo/download/?acc=GSE183852&format=file&file=GSE183852%5FDCM%5FIntegrated%2ERobj%2Egz). From the snRNAseq data, we created cell type-specific gene expression profiles for 11 different cell types using an adapted approach from sc-linker^39^. We then used stratified LD score regression^100^, with CC-GWAS as input and using variant-to-gene mapping strategies from sc-linker^39^, to compute heritability enrichment statistics for each cell type. Similar analyses were then performed for each cell state. Analyses were one-sided, and multiplicity correction was performed using the Bonferroni. Details are provided in the **Supplementary Note**.

#### Functional enrichment

We used the g:Profiler platform^40^ (v. February 2025) to test for enrichment of gene sets from several predefined sources for genes curated from CC-GWAS and CC-MTAG. The g:Profiler algorithm uses one-sided Fisher’s exact tests to test for enrichment of a prespecified list of genes across many gene sets, and subsequently adjusts one-sided P values for multiple testing while taking into account the correlation between gene sets (g:SCS method76). First, we tested all genes from CC-GWAS, then genes unique to CC-GWAS or CC-MTAG. Since our prioritized genes may have been preselected towards genes with high cardiac expression (that is, through gene features learnt by PoPS), we performed sensitivity analyses using the nearest-gene annotation for loci identified in the CC GWAS and CC MTAG analyses; to this end, we used the get_nearest_gene() function from the *gwasRtools* package (v0.1.0; available via GitHub: lcpilling/gwasRtools). (Extended Data Figure 6, **Supplementary Table 2, Supplementary Note**)

### PheWAS

To explore potential pleiotropic effects of lead variants at novel loci, we performed a phenome-wide association study (PheWAS) using data from the Cardiovascular Disease Knowledge Portal (CVDKP). We queried lead variants from 17 novel loci identified in CC GWAS or CC MTAG (Extended Data Figure 4, **Supplementary figures 4a–s; Supplementary Table 11**) and one locus from shared meta-analysis (Figure 5a,c; **Supplementary Table 13**) and assessed their associations across a broad spectrum of traits.

### Drugability predictions

To assess the therapeutic potential of prioritized genes, we systematically annotated druggability using both the Open Targets Platform^51^ (queried in April 2025) and DrugnomeAI^52^. **(Supplementary Note)**

### Replication and validation analyses

#### Independent cohorts

To replicate the novel findings, we performed analyses on an independent dataset of HCM and DCM cases, combined into a single joint whole-genome sequencing dataset. HCM cases were recruited from the Sarcomeric Human Cardiomyopathy Registry (SHaRe). The registry’s structure and initial findings have been detailed by Ho et al. (2018)^101^. DCM cases were predominantly recruited from GoDCM, an effort that combines patients from several clinical centers across the UK. Further details are presented in the **Supplementary Note.**

#### CC-GWAS replication

The case–case GWAS replication analysis was performed in Hail using an additive genetic model with SNP dosage. HCM cases were treated as “cases” and DCM cases as “controls.” Covariates included sex and the top 20 principal components. Association statistics were reported as log-odds ratios per effect allele with corresponding standard errors and p-values.

#### Validation of the novel case-cases loci.

To assess replication of novel lead SNPs from the discovery CC-GWAS and CC-MTAG analyses, we extracted corresponding variants from the individual-level case–case GWAS (lifted to GRCh38 and allele-harmonized), using MTAG estimates when SNPs appeared in both analyses. Replication was evaluated using direction concordance (binomial test), one-sided association lookups in the discovery-predicted direction, and effect size concordance via correlation and regression of betas and Z-scores between the discovery and validation datasets.

#### Validation of the novel shared locus.

To validate the association of the lead variant in locus from shared-effect meta analysis, we compared allele frequencies in QC-passed HCM and DCM cases against non-Finnish European controls from gnomAD (v4.1) under an additive model using one-sided Fisher’s exact tests.

### Polygenic score analyses

#### Creating and testing polygenic scores for cardiomyopathy

We then aimed to construct polygenic scores (PGS) from our cardiomyopathy GWAS data. To this end, we used the recently described SBayesRC algorithm (https://github.com/zhilizheng/SBayesRC)^57^. SBayesRC improves polygenic prediction by leveraging functional annotations and substantially increasing genomic coverage compared to many other methods. We used SBayesRC (v0.2.6) to build a PGS from the DCM MTAG summary statistics (PGS-DCM), a PGS from the HCM MTAG summary statistics (PGS-HCM), and finally a novel PGS from our CC-MTAG summary statistics (PGS-CC). When running SBayesRC, we used functional annotation data for 8,140,664 SNPs from the Baseline-LD v2.2 model^102^, which includes variant-level information such as enhancer or promoter region status, with corresponding annotation-based weights. The LD reference we used was constructed from imputed SNPs in 347,800 individuals of European ancestry from the UK Biobank^57^.

We then applied the three PGSs to the All of Us Research Program (AoU). AoU is a cohort study enrolling participants from across the United States, with an emphasis on participants classically underrepresented in genetics research^103^. Whole genome sequencing data were available for over 410,401 participants after quality control, of which 76.8% had complete electronic health record (EHR) linkage (version 8 of AoU). In our analyses, we restricted to samples with high-quality genomic data and EHR data available. Detailed QC has been described elsewhere.^66^ We further excluded individuals residing within Massachusetts (as these may overlap with samples included in our GWAS^104^).

These AoU participants were scored for each of the PGSs using the ‘--score’ function, implemented in PLINK2. Before running analyses, to account for ancestral structure in PGS distributions, we first regressed out the first 20 ancestral principal components (PCs) from the PGS values, resulting in a residualized PGS score for each individual. We then corrected for the variance within the residualized PGS due to ancestral structure, by dividing the residualized PGS by the PC-predicted standard deviation of the residualized PGS^105,106^. Finally, we standardized the ancestry-corrected PGSs to mean 0 and unit variance. Subsequently, we extracted individuals with genetically-inferred European ancestry, leaving us with around 147,000 European ancestry samples, of which 1,053 had DCM and 562 had HCM (**Supplementary Tables 14–15**).

We then assessed whether each of the PGSs could discriminate between various cardiomyopathy cases and controls, as well as among themselves. Notably, we used logistic regression models to discriminate i) DCM cases from controls, ii) HCM cases from controls, and finally iii) DCM cases from HCM cases. All logistic regression models were adjusted for the first 20 principal components of ancestry; age and sex were included as covariates except in univariate models. From the regression models we computed several performance metrics^15^, including i) the (log-)odds ratio per standard deviation increase in PGS, ii) the area under the receiver-operator-characteristics curve (univariate model), iii) the area under the precision-recall curve (univariate model), iv) the improvement in Nagelkerke pseudo-R^2^, and v) the improvement in liability-scale R^2^. Correcting for the number of tests, we considered results with *P* < 0.05 ((3 × 3)) = 0.00556 significant. In all PGS analyses, hypothesis tests were two-sided.

In addition to analyses in the All of Us cohort, we performed similar PGS analyses in the replication cohort comprising HCM samples from SHaRe and DCM samples from GoDCM. In this dataset, we tested only PGS-DCM, PGS-HCM, and PGS-CC for discriminating DCM cases from HCM cases. Further details are presented in the **Supplementary Note.**

#### Creating and testing polygenic scores for cardiometabolic and MRI traits

We then asked whether the polygenic components underlying cardiometabolic and intrinsic myocardial traits showed consistent or opposing directionality in DCM and HCM. To this end, using similar methods as described above, we used the SBayesRC algorithm to construct PGS for several cardiometabolic and cardiac MRI traits^66^. We tested these for association with DCM and HCM status in the All of Us Research Program^58^. Notably, we created PGS for cardiometabolic traits, including AF^64,67^, CAD^68^, BMI^69^, SBP^67,70^, DBP^71^, and CRP^72^, and for cardiac MRI traits, including LV concentricity (LVconc), negative value of circumferential strain (-Ecc), LV ejection fraction (LVEF), and indexed end-systolic volume (LVESVi)^16^. Correcting for the number of tests, we considered results with *P* < 0.05 ((10 × 2)) = 0.0025 significant, while *P*<0.05 was considered suggestive. Hypothesis tests were two-sided.

## Extended Data

## Supplementary Material

Supplementary Files

This is a list of supplementary files associated with this preprint. Click to download.

• Supplementarymaterials11Dec2025.pdf

• Supplementarytables11Dec2025.xlsx

## Data Availability

Summary statistics from our case-case GWAS, case-case MTAG, and shared-effect meta-analysis will be made publicly available for download through the Cardiovascular Disease Knowledge Portal, upon acceptance of the article. The original DCM GWAS and MTAG summary statistics, used in the present study, are publicly available for download from the Cardiovascular Disease Knowledge Portal (https://cvd.hugeamp.org/downloads.html); the original HCM GWAS and MTAG summary statistics, as well as GWAS summary statistics for cardiac MRI traits, are available for download through the GWAS Catalog (accession IDs GCST90435254–GCST90435267). PGS scoring files for our new DCM, HCM, and case-case PGSes will be made available through PGScatalog, upon acceptance of the article. Access to individual-level phenotypic and genetic data from the All of Us Research Program is currently available to researchers from verified institutions worldwide, through the All of Us Researcher Workbench—a cloud-based computing platform (https://www.researchallofus.org/register/). Access to individual-level phenotypic and genomic data for the GoDCM and SHaRe datasets is not publicly availabledue to the data’s restrictive or sensitive nature. The snRNAseq data from Reichart et al. were downloaded from GEO (https://www.ncbi.nlm.nih.gov/geo/download/?acc=GSE183852&format=file&file=GSE183852%5FDCM%5FIntegrated%2ERobj%2Egz).

## Software and code availability statement

All computational analyses were performed using publicly available software, as detailed below. Genotype processing, quality control, imputation, GWAS, fine-mapping, gene prioritization, local genetic correlation, tissue enrichment, and polygenic scoring pipelines were implemented through custom Bash, Python, and R workflows, which are available upon reasonable request. Example of the major scripts available in GitHub (https://github.com/drkramarenko/shared_opposing_mechanisms_cmp) **LD Score Regression (LDSC)** v1.0.1 (https://github.com/bulik/ldsc) was used to compute SNP-based heritability, global genetic correlations, and partitioned heritability estimates. Multitrait analyses were performed using **MTAG** v1.0.8 (https://github.com/JonJala/mtag) in a dedicated Python 2.7 environment. Case–case analyses were conducted using **CC-GWAS** v0.2 (https://github.com/wouterpeyrot/CCGWAS). Local genetic correlation analyses were performed using **LAVA** v1.1 (https://github.com/josefin-werme/LAVA) with reference LD from the 1000 Genomes and UK Biobank. Fine-mapping of all GWAS datasets was performed using **SuSiE** (susieR) v0.12.35 (https://github.com/stephenslab/susieR) within UK Biobank–derived LD blocks. Variant-to-gene links, gene scores, and effector gene prioritization were performed using **FUMA** v1.6.1 (https://fuma.ctglab.nl/), **MAGMA** v1.10 (https://ctg.cncr.nl/software/magma), **PoPS** v0.2 (https://github.com/FinucaneLab/pops), and **FLAMES** v1.1.1 (https://github.com/Marijn-Schipper/FLAMES). All locus definitions, credible set processing, and cross-study harmonization were implemented using custom R scripts .Gene-set and pathway enrichment analyses were performed using **FUMA** v1.6.1 and **g:Profiler** (release 2025–02; https://biit.cs.ut.ee/gprofiler/). Dimensionality reduction and REVIGO-based redundancy filtering were conducted using **REVIGO** (http://revigo.irb.hr/), with custom integration scripts in R.Druggability annotations were obtained using the **Open Targets Platform** (release April 2025; https://platform.opentargets.org) and **DrugnomeAI** v1.0 (https://astrazeneca-cgr-publications.github.io/DrugnomeAI). Polygenic scores were generated with **SBayesRC** (v0.2.6) (https://github.com/zhilizheng/SBayesRC) and **PLINK2** (https://www.cog-genomics.org/plink/2.0/; various versions from February 2025 release onwards). Code for running SBayesRC on the all-ancestry AF summary statistics is provided (https://github.com/poeyahay/AFib_PGS/blob/main/SBayesRC/SBRC_Run.sh). Using the cell type-specific gene expression profiles, we then performed heritability enrichment analyses with the *sc-linker* pipeline (https://github.com/kkdey/GSSG) on preprocessed snRNA-seq data from Reichart et al., 2022.

Downstream statistical analyses, figure generation (including locus plots, LAVA Manhattan plots, PGS forest plots, druggability volcano plots) were conducted in **R** v4.3.1 or higher.
